# Impact of campaign-style delivery of routine vaccines: a quasi-experimental evaluation using routine health services data in India

**DOI:** 10.1093/heapol/czab026

**Published:** 2021-03-18

**Authors:** Emma Clarke-Deelder, Christian Suharlim, Susmita Chatterjee, Logan Brenzel, Arindam Ray, Jessica L Cohen, Margaret McConnell, Stephen C Resch, Nicolas A Menzies

**Affiliations:** Department of Global Health and Population, Harvard T. H. Chan School of Public Health, 665 Huntington Avenue, Boston, MA 02115 USA; Center for Health Decision Science, Harvard T. H. Chan School of Public Health, 718 Huntington Avenue, Boston MA 02115, USA; Management Sciences for Health, 200 Rivers Edge Dr, Medford MA 02155, USA; Research Department, George Institute for Global Health, 308-309 Elegance Tower, Plot No. 8, Jasola District Centre, New Delhi -110025, India; Department of Medicine, University of New South Wales, 18 High Street, Kensington, New South Wales, 2052, Australia; Bill & Melinda Gates Foundation, 500 5th Ave N, Seattle, WA 98109, USA; Bill & Melinda Gates Foundation, Capital Court, The 5th Floor, Olof Palme Marg, Munirka, New Delhi, Delhi 110067, India; Department of Global Health and Population, Harvard T. H. Chan School of Public Health, 665 Huntington Avenue, Boston, MA 02115 USA; Department of Global Health and Population, Harvard T. H. Chan School of Public Health, 665 Huntington Avenue, Boston, MA 02115 USA; Center for Health Decision Science, Harvard T. H. Chan School of Public Health, 718 Huntington Avenue, Boston MA 02115, USA; Department of Global Health and Population, Harvard T. H. Chan School of Public Health, 665 Huntington Avenue, Boston, MA 02115 USA; Center for Health Decision Science, Harvard T. H. Chan School of Public Health, 718 Huntington Avenue, Boston MA 02115, USA

**Keywords:** Vaccination, quasi-experimental design, health services research

## Abstract

The world is not on track to achieve the goals for immunization coverage and equity described by the World Health Organization’s Global Vaccine Action Plan. Many countries struggle to increase coverage of routine vaccination, and there is little evidence about how to do so effectively. In India in 2016, only 62% of children had received a full course of basic vaccines. In response, in 2017–18 the government implemented Intensified Mission Indradhanush (IMI), a nationwide effort to improve coverage and equity using a campaign-style strategy. Campaign-style approaches to routine vaccine delivery like IMI, sometimes called ‘periodic intensification of routine immunization’ (PIRI), are widely used, but there is little robust evidence on their effectiveness. We conducted a quasi-experimental evaluation of IMI using routine data on vaccine doses delivered, comparing districts participating and not participating in IMI. Our sample included all districts that could be merged with India’s 2016 Demographic and Health Surveys data and had available data for the full study period. We used controlled interrupted time-series analysis to estimate the impact of IMI during the 4-month implementation period and in subsequent months. This method assumes that, if IMI had not occurred, vaccination trends would have changed in the same way in the participating and not participating districts. We found that, during implementation, IMI increased delivery of 13 infant vaccines, with a median effect of 10.6% (95% confidence interval 5.1% to 16.5%). We did not find evidence of a sustained effect during the 8 months after implementation ended. Over the 12 months from the beginning of implementation, we estimated reductions in the number of under-immunized children that were large but not statistically significant, ranging from 3.9% (−6.9% to 13.7%) to 35.7% (−7.5% to 77.4%) for different vaccines. The largest effects were for the first doses of vaccines against diphtheria-tetanus-pertussis and polio: IMI reached approximately one-third of children who would otherwise not have received these vaccines. This suggests that PIRI can be successful in increasing routine immunization coverage, particularly for early infant vaccines, but other approaches may be needed for sustained coverage improvements.

**KEY MESSAGES**

- Intensified Mission Indradhanush, a campaign-style effort to increase routine immunization coverage in India, significantly increased delivery of infant vaccines during the implementation period.
- The effect was largest for the first doses of polio and diptheria-tetanus-pertussis vaccines, suggesting that this strategy was successful in reaching ‘zero dose children’.
- We did not find evidence of a sustained effect after program implementation ended.
- ‘Periodic intensification of routine immunization’ may be an effective strategy for reaching zero dose children, increasing immunization coverage and improving equity in the short-term.

## Introduction

Despite significant investments in improving immunization coverage, the world is not on track to achieve the goals set by the World Health Organization (WHO)’s Global Vaccine Action Plan for 2011–20 (WHO, 2013). Global coverage of the third dose of diphtheria-tetanus-pertussis (DTP) vaccine, a measure of routine immunization system performance, stagnated between 2011 and 2018 (World Health Organization, 2018). In India in 2016, only 62% of children received a full course of basic vaccines, and state-level coverage ranged from 35% to 91% (National Family Health Survey (HFHS-4), 2015).

There is little rigorous evidence about how to effectively improve routine immunization coverage (Oyo-Ita *et al.*, 2016; Munk *et al.*, 2019). One widely applied strategy—‘periodic intensification of routine immunization’ (PIRI)—adapts techniques from mass immunization campaigns and applies them to the delivery of routine vaccines (John Snow International, 2009; World Health Organization, 2016). Mass immunization campaigns have been reported to achieve high vaccine coverage (Shearer *et al.*, 2017) of a single or small number of vaccines, but there is little robust evidence on the effectiveness of campaign-like approaches for delivering the full schedule of routine vaccines. While campaign-like approaches can reach many people, some fraction of these individuals may have been reached by routine services anyway. Delivery volumes reported by campaigns may therefore overestimate the net change in coverage.

In 2017–18, the Government of India implemented a nationwide effort to improve routine immunization coverage and equity, via a campaign-like initiative called Intensified Mission Indradhanush (IMI). IMI was designed to (1) increase coverage of routine vaccines for infants under two and pregnant women in selected low-performing districts and (2) sustain these gains by raising public awareness of routine immunization and strengthening routine planning (Ministry of Health & Family Welfare, Government of India, 2017). IMI was one of the largest ever applications of the PIRI strategy.

In this study, we conducted a quasi-experimental evaluation of IMI. Using a controlled interrupted time-series approach, we estimated the impact of IMI on vaccine delivery, coverage and the number of unvaccinated children for 15 vaccines in the routine immunization schedule.

## Materials and methods

### Study setting

India’s immunization program serves a target population of ∼27 million newborns and 30 million pregnant women annually (Gurnani *et al.*, 2018). Vaccinations are provided through public health facilities and community-based outreach sessions, with an estimated 9 million immunization sessions held annually (Chatterjee *et al.*, 2016). The routine schedule includes 25 childhood vaccines and two vaccines for pregnant women (Table 1). Vaccines are administered according to a schedule by age, but some vaccines can be administered after the first recommended age under catch-up vaccination policies (Frequently Asked Questions on Immunization \(For Health Workers & Other Front-line Functionaries\) [Internet], 2017).

**Table 1:** National immunization schedule for children and pregnant women in India

Time point in schedule	Vaccine	Upper age limit
Birth	BCG	Up to 1 year
Birth	OPV0	Up to 15 days after birth
Birth	HepB0	Up to 24 h after birth
6 weeks	Penta 1 (containing DTP1)	Up to 1 year
6 weeks	OPV1	Up to 5 years
6 weeks	IPV1	Up to 1 year
6 weeks	Rota1	Up to 1 year
6 weeks	PCV1	Up to 1 year
10 weeks	Penta 2 (containing DTP2)	Any age (as long as first dose is given by 1 year)
10 weeks	OPV2	Up to 5 years
10 weeks	Rota2	Any age (as long as first dose is given by 1 year)
10 weeks	PCV2	Any age (as long as first dose is given by 1 year)
14 weeks	Penta 3 (containing DTP3)	Any age (as long as first dose is given by 1 year)
14 weeks	OPV3	Up to 5 years
14 weeks	IPV2	Any age (as long as first dose is given by 1 year)
14 weeks	Rota3	Any age (as long as first dose is given by 1 year)
14 weeks	PCV3	Any age (as long as first dose is given by 1 year)
9 months	JE1	Up to 15 years
9 months	M1	Up to 5 years
16 months	DTP-b	Up to 7 years
16 months	M2	Up to 5 years
16 months	OPV-b	Up to 5 years
16 months	JE2	Up to 15 years
5–6 years	DTP-b2	Up to 7 years
10 years	TT	Any age
As soon as pregnancy is confirmed	TT1	During labour
During pregnancy, 4 weeks after TT1	TT2	During labour
During pregnancy, if received 2 TT doses in a pregnancy within the past 3 years	TTb	During labour

*Notes*: Table shows the schedule of vaccines provided for free by India’s national immunization program to infants (16 months and under) and pregnant women. The vaccines provided include: Bacillus Calmette-Guérin vaccine (BCG), oral polio vaccine (OPV), Hepatitis B vaccine (HepB), pentavalent vaccine (Penta), oral polio vaccine (OPV), rotavirus vaccine (Rota), pneumococcal conjugate vaccine (PCV), Japanese Encephalitis vaccine (JE), measles vaccine (M) and tetanus toxoid vaccine (TT). The number next to the vaccine indicates the dose number in the vaccination schedule. The letter ‘b’ indicates a booster shot. The pentavalent vaccine contains protection against diphtheria, tetanus, pertussis, *Haemophilus Influenzae* B and Hepatitis B. It replaced three doses of the diphtheria-tetanus-pertussis (DTP1, DTP2 and DTP3) vaccine in India’s immunization schedule in 2011, though DTP was still used in some areas at the beginning of the study period.

Many children and pregnant women do not receive the full schedule of vaccines: in 2016, 78% of children aged 12–23 months had received three doses of the diphtheria-tetanus-pertussis (DTP) vaccine, 73% had received three doses of the polio vaccine, and 62% had received a full course of basic vaccines (National Family Health Survey HFHS-4, 2015). Among women who had given birth in the previous 5 years, 89% had been protected against neonatal tetanus for their most recent birth (National Family Health Survey HFHS-4, 2015). Immunization coverage is lower among children in lower-income households, in households with lower rates of parental education, and in households where the mother did not receive the recommended number of antenatal or postnatal care visits (Sissoko *et al.*, 2014). Reasons for under-immunization include supply-side issues such as inaccessibility of vaccination services (Francis *et al.*, 2018) and under-staffing of health facilities (Vashishtha, 2012) and demand-side issues such as low awareness (Francis *et al.*, 2018) and anti-vaccine sentiment (Laxminarayan and Ganguly, 2011). In a 2008 household survey, the most common reason for under-vaccination (reported by caregivers) was unawareness of the need for vaccination (45%) (Francis *et al.*, 2018). Religious beliefs may also play an important role: a 2016 multi-country survey of vaccine confidence found that, in India, 4.9% of respondents disagreed with the statement that ‘vaccines are important for children to have’, but 20.8% of respondents disagreed with the statement that ‘vaccines are compatible with my religious beliefs’ (Larson *et al.*, 2016).

In recent years, in an effort to increase coverage, the Government of India implemented a series of campaign-like interventions called Mission Indradhanush (MI). These interventions fell short of their objectives, leading to the design and implementation of IMI (Gurnani *et al.*, 2018).

### Intervention

The Government of India implemented IMI from October 2017 through January 2018 as part of the Pro-Active Governance and Timely Implementation (PRAGATI) initiative, a set of programs prioritized by the office of the Prime Minister. Districts with weak immunization performance (<70% estimated DTP3 coverage, or >13 000 children missing DTP3 in the previous year) were included, and additional districts added based on requests from states (Gurnani *et al.*, 2018). In total, 187 districts and urban areas were included.

IMI implementation began with door-to-door surveys to identify under-immunized children. District-level micro-plans were then developed to determine the location of IMI vaccination sites and ensure supply availability. Site selection focused on areas with low coverage, with particular emphasis on urban slums and nomadic populations. Social mobilization campaigns, led primarily by community health workers, were conducted to raise awareness. Finally, immunization sessions were conducted for seven consecutive days per month during implementation. Sessions were conducted by auxiliary nurse-midwives (ANMs), who left their postings in periphery health facilities to deliver vaccines and other health services at the selected locations.

### Data sources

Outcome data at the district-month level were extracted from India’s Health Management Information System (HMIS), which compiles service delivery data reported by health facilities. We used data on vaccine doses delivered from October 2015 through September 2018, encompassing 2 years before the start of IMI and 1 year after. We used data on 13 vaccines for children and 2 for pregnant women: Hepatitis B birth dose (HepB0), Bacillus-Calmette-Guérin (BCG), 4 doses of diphtheria, tetanus, and pertussis-containing vaccines [DTP1, DTP2, DTP3 and DTP booster (DTPb)], 5 doses of oral polio vaccine [OPV0, OPV1, OPV2, OPV3 and OPV booster (OPVb)], 2 doses of measles-containing vaccines (M1 and M2); and the first and second dose (or booster) of tetanus toxoid vaccine (TT1 and TT2) for pregnant women. We also used HMIS data on the number of immunization sessions held per district-month. We excluded the Japanese encephalitis vaccine because it is only delivered in endemic areas. We excluded rotavirus, pneumococcal and inactivated polio vaccines because they were recently introduced and not available for the full study period. We excluded vaccines for children over age two (a second DTP booster and tetanus toxoid) because IMI primarily targeted children under two and pregnant women. While we included HepB0 and OPV0, we did not expect large effects for these vaccines because the catch-up period is limited to 24 h and 15 days post-birth, respectively.

To identify districts included in IMI, we used publicly available documents from the Indian Universal Immunization Program (Intensified Mission Indradhanush: Operational Guidelines [Internet]., 2017). We also extracted covariate data from India’s 2015–16 Demographic and Health Surveys (DHS), summarized at the district level (National Family Health Survey HFHS-4, 2015). This included vaccine coverage and urbanization (percent of children under five living in an urban area). Finally, we used World Bank estimates of India’s 2017 population size, fertility rate and neonatal mortality rate to generate estimates of the target population size for different vaccines (World Bank, 2017).

### Sample

The study sample included all districts in India meeting two criteria: (1) they had available HMIS data for the full study period and (2) these data could be merged with DHS data. Out of 187 treated districts, there were 4 districts missing from the HMIS dataset, and we omitted an additional 4 districts because they experienced complex administrative changes during the study period and therefore could not be merged with the DHS data. Out of 549 control districts, there were 21 districts missing from the HMIS dataset, we omitted an additional 87 districts (all in three states: West Bengal, Telangana and Chattisgarh) because they experienced complex administrative changes during the study period and could not be merged with the DHS data, and we omitted two districts because they had incomplete time-series data for the study period. Overall, the omissions represented 2% of live births in the HMIS system in 2017 in treated districts, and 17% in control districts. The omission of 87 control districts from three states is unlikely to affect our findings, as two of these states had no treated districts (Telangana and Chattisgarh) and one had only one treated district (West Bengal). Further details of the sampling procedure are included in Supplementary Table S1.

### Statistical analysis

We conducted a comparative interrupted time-series (CITS) analysis. This quasi-experimental method accounts for baseline differences between treated and control groups, for time trends that would have occurred in the absence of the intervention and for exogenous shocks that could have affected the outcome trend in both the treatment and control group (Jandoc *et al.*, 2015; Bernal *et al.*, 2018). CITS differs from single interrupted time-series in that the outcome trend is modelled in a control group as well as a treatment group in order to account for any factors, apart from the treatment, that might have changed the outcome trend in both groups (Bernal *et al.*, 2019). CITS differs from difference-in-differences analysis in that it does not require an assumption of parallel trends in the absence of the intervention. Our analysis assumed that, in the absence of IMI, deviations from past trends in vaccine delivery would have been the same for treated and control districts. This approach accounts for the possibility that IMI could displace vaccine delivery that would have occurred even in the absence of the program. If not accounted for in the analysis, this displacement could produce overestimates of the impact of IMI on vaccine delivery.

We modelled time trends in district-level vaccination volume (doses delivered) using generalized linear models with quasi-Poisson distributed outcomes and a log link, in order to appropriately model count data with over-dispersion (Novoa *et al.*, 2011; Lopez Bernal *et al.*, 2013). We defined the ‘pre-intervention’ period as October 2015 through September 2017, the ‘implementation’ period as October 2017 through January 2018 and the ‘post-implementation’ period as February 2018 through September 2018. Our models included dummy variables to model intercept changes on 1 October 2017 (the start of IMI), 1 February 2018 and 1 June 2018. We therefore measured the impact of IMI on vaccine delivery during the 4-month implementation period and two 4-month post-implementation periods. By including two post-implementation periods, we assessed whether immunization delivery volumes returned to or dipped below their pre-intervention levels after IMI implementation ended. From this analysis, we estimated the impact of IMI during the implementation period, as well as the net impact over the full year following the beginning of implementation.

We assessed whether IMI was more effective in districts with lower coverage or higher levels of urbanization (because these were focus areas of the program) by including interaction terms in our regression models. We also included calendar month fixed effects to adjust for seasonality and district fixed effects to absorb variation in the initial level of vaccination volume. We used Newey–West standard errors to adjust for potential serial autocorrelation (Newey and West, 1987).

Because the treatment effect in CITS is captured by multiple coefficients, it is common practice to generate interpretable results by making predictions from fitted models (Wagner *et al.*, 2002). Following this practice, we estimated vaccination volume in the treated districts if IMI had not occurred and compared this to the observed vaccination volume under IMI. We estimated the incremental vaccination volume attributable to IMI by projecting model results to the full set of treated districts with covariates fixed to their true values. To describe how the estimated treatment effect varied by district characteristics, we generated treated effect estimates from models fixing the values of covariates to their 25th and 75th percentiles in the treated districts. We estimated equal-tailed 95% confidence intervals (95% CIs) for all effects by estimating the treatment effects over 1000 simulations from the fitted models and calculating the 2.5th and 97.5th quantiles of the effect distributions (Zhang *et al.*, 2009).

To estimate the impact of IMI on coverage in the treated districts, we divided our estimates of incremental doses delivered by estimates of the target population size. Since data on the target population size were not available for 2017, we estimated the target population size for HepB, OPV0, BCG, TT1 and TT2 as the number of live births in 2017, calculated as fertility rate multiplied by population size. We estimated the target population size for DTP1–3, OPV1–3 and M1–2 as the number of surviving infants in 2017, calculated by multiplying live births by one minus the neonatal mortality rate. To estimate the percent reduction in unvaccinated children, we divided our estimates of incremental doses delivered by estimates of the number of children who would not have been reached if IMI had not occurred, if the assumptions of our CITS were met and coverage in the treated districts had remained the same as in 2016 in the counterfactual in which IMI was not implemented. We estimated the number of unvaccinated children by multiplying the target population size by one minus coverage in 2016. We calculated 95% CIs for this analysis using the same approach as above.

To examine mechanisms for the treatment effect, we measured the impact of IMI on the number of immunization sessions held (including both routine and IMI sessions), and the ratio of incremental doses delivered to incremental immunization sessions held.

All statistical analyses were conducted in R.

### Sensitivity analysis

We examined the robustness of our results to different model specifications. First, we conducted a single interrupted time-series analysis. This method uses data only from the treated districts and relies on the assumption that the trend in vaccination volume in the treated districts would have been maintained during the treatment period if the intervention had not occurred. Second, we conducted matched analyses using a subset of the study sample. We used coarsened exact matching (Iacus *et al.*, 2012) to match treatment and control districts on geographic location, baseline coverage, urbanization level and participation in the earlier MI program. This method controls for potential confounding by the matching variables. In the case of CITS, confounding could occur if some underlying differences between the treatment and control districts were associated with the change in the outcome trend during the study period. Finally, we examined the possibility of spill over effects by conducting an analysis in which the control group was restricted to untreated districts that do not share borders with treated districts.

Supplementary Appendix SA provides detailed analytic methods.

### Role of the funding source

Employees of the funder (L.B. and A.R.) participated as scientific collaborators. The corresponding author made the final decision to submit the paper for publication.

## Results

### Impact of IMI on vaccination volume

**Figure 1:**
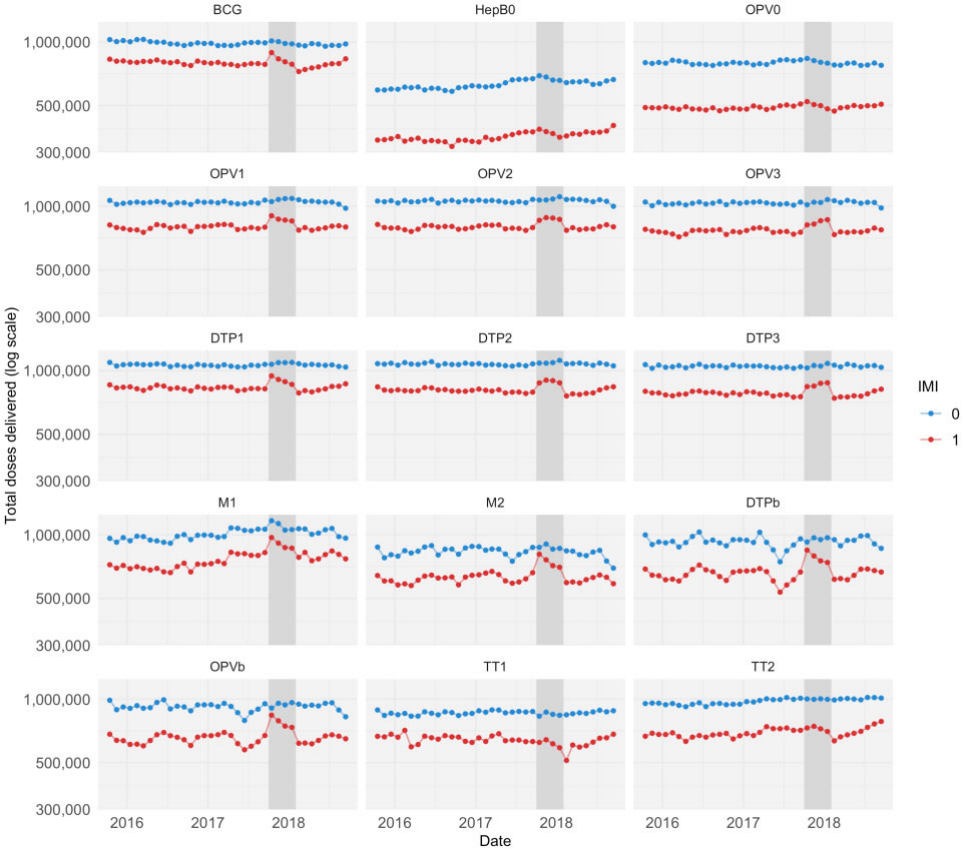
Time trends in vaccination volume in treated and untreated districts, 2 years before and 1 year after the start of IMI implementation. *Notes*: This figure shows time trends in total vaccine doses delivered in treated (red) and untreated (blue) districts in the sample. Trends are shown for the Bacillus Calmette-Guérin (BCG) vaccine, the birth dose of the Hepatitis B vaccine (HepB0), four doses of DTP-containing vaccine (delivered as part of the pentavalent vaccine in recent years), five doses of the oral polio vaccine (OPV) and two doses of tetanus toxoid vaccine (TT). All vaccines shown are given to infants, apart from the tetanus toxoid vaccine, which is given to pregnant women. The dark grey bar indicates the 4-month period of IMI implementation, from October 2017 through January 2018. The 8-month period to the right of the dark grey bar is the ‘post-implementation’ period, which is included in our analyses of the impact of IMI over a 1-year period. Raw counts of doses delivered were adjusted for seasonality by subtracting calendar month fixed effects estimated using linear regression models.

**Figure 2:**
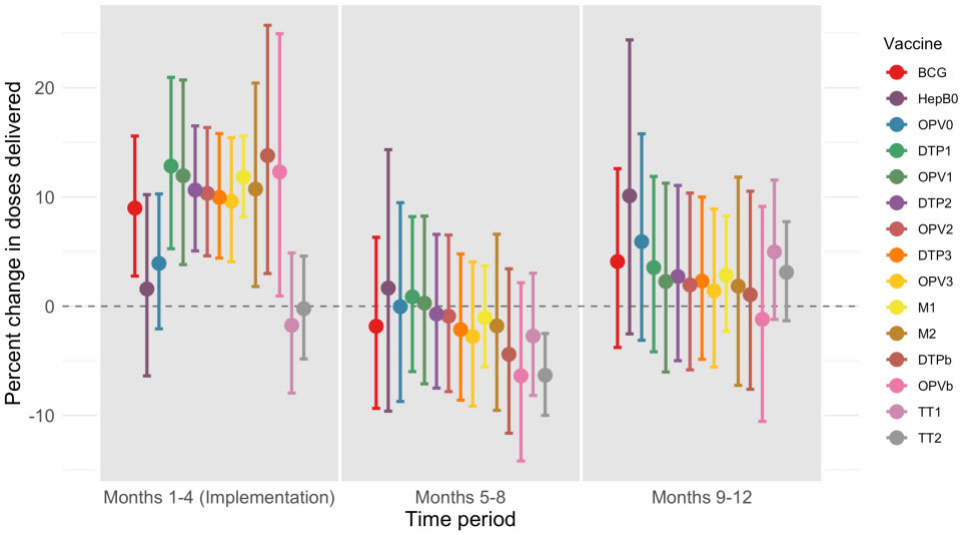
Regression results: percent change in doses delivered in treated districts during three 4-month periods. *Notes*: This figure shows estimates of the effect of IMI on doses delivered, measured as a percent change in doses delivered in each of three time periods [(1) during implementation (Months 1–4); (2) the 4 months following implementation (Months 5–8); and (3) the 4 months after that (Months 9–12). Covariate values (urbanization and baseline coverage) are fixed at their mean values. To show percent change, we first exponentiate regression coefficients (because models are fit with a log link function), then subtract one and multiply by 100. To calculate 95% confidence intervals on the same scale, we first calculate 95% confidence intervals on the log scale using assumptions from the normal distribution, and then exponentiate the interval bounds, subtract one and multiply by 100.

During the 4-month implementation period, IMI had a positive estimated impact on delivery volume for all infant vaccines in the study (Figures 1 and 2). Figure 1 shows the trends over time in vaccination volume in the treated and untreated districts. Vaccination volumes increased visibly during the implementation period in the treated districts but not in the control districts. Using CITS, we estimate that IMI increased infant vaccination volume in the treated districts by between 1.6% (HepB0; 95% CI −6.4 to 10.2) and 13.8 (DTPb; 3.0 to 25.7), with a median of 10.6% across the different vaccines in the study. The point estimates for DTP1 and DTP3 were 12.8% (95% CI 5.3 to 21.0) and 10.0% (95% CI 4.4 to 15.8), respectively. For HepB0 and OPV0, while the point estimate for the treatment effect was positive, the estimate was not statistically significant. In addition, there was no statistically discernable impact of IMI on the delivery of vaccines for pregnant women (median −0.9% change in vaccination volume).

The estimated effect of IMI waned after implementation ended. With the exception of TT2, there was no statistically discernable impact of IMI on delivery of any antigen during the period from February to May 2018 or the period from June to September 2018. Point estimates for all estimated effects were modestly negative from February to May 2018 (median value −1.8%), with the exception of OPV0, DTP1 and OPV1, for which the point estimates were positive. Point estimates for all estimated effects were positive from June to September 2018 (median value 2.7%), with the exception of OPVb, for which the estimated effect was negative, but none of the effects was statistically significant.

Over the full year from the beginning of implementation, we estimated positive impacts of IMI on the number of doses delivered of all infant vaccines and TT1, and a negative impact on doses delivered of TT2 (Table 2). None of these estimates were statistically significant. We estimated that IMI reached between 148 000 and 491 000 additional infants with each of the vaccines in the study. However, the 95% CIs included the possibility that IMI led to a reduction in the number of children reached across all vaccines during the year. We estimated that IMI reached 6000 additional pregnant women with TT1, with a 95% CI from −393 000 to 398 000. Finally, we estimated that IMI reduced the number of women vaccinated with TT2 by 102 000, with a 95% CI ranging from a reduction of 441 000 to an improvement of 215 000.

**Table 2:** Estimated impact of IMI on number of children reached, coverage in the treated districts, and unvaccinated children in India over 1 year (October 2017 through September 2018)

	(1) Number of additional children reached (thousands) (95% CI)	(2) Percentage point increase in coverage in treated districts (95% CI)	(3) Percentage of unvaccinated children in the treated districts reached through IMI (95% CI)
BCG	331	3.9	31.5
	(−282 to 883)	(−3.3 to 10.4)	(−26.8 to 83.9)
HepB0	148	1.7	3.9
	(−263 to 521)	(−3.1 to 6.1)	(−6.9 to 13.7)
OPV0	136	1.6	5.2
	(−299 to 500)	(−3.5 to 5.9)	(−11.5 to 19.2)
DTP1	491	5.9	35.7
	(−100 to 1033)	(−1.2 to 12.5)	(−7.5 to 77.4)
OPV1	396	4.8	33.1
	(−221 to 969)	(−2.7 to 11.7)	(−18.5 to 81.2)
DTP2	342	4.1	19.7
	(−187 to 832)	(−2.3 to 10.1)	(−10.7 to 47.8)
OPV2	287	3.5	17.2
	(−252 to 823)	(−3.0 to 9.9)	(−15.1 to 49.4)
DTP3	253	3.1	10.0
	(−274 to 733)	(−3.3 to 8.8)	(−10.9 to 29.0)
OPV3	192	2.3	6.9
	(−318 to 670)	(−3.8 to 8.1)	(−11.5 to 24.3)
M1	377	4.5	16.9
	(−23 to 771)	(−0.3 to 9.3)	(−1.0 to 34.7)
DTPb^a^	233	2.8	NA
	(−474 to 857)	(−5.7 to 10.3)	
M2^a^	247	3.0	NA
	(−372 to 793)	(−4.5 to 9.6)	
OPVb^a^	79	1.0	NA
	(−662 to 773)	(−8.0 to 9.3)	
TT1	6	0.1	0.7
	(−393 to 398)	(−4.6 to 4.7)	(−47.2 to 49.9)
TT2	−102	−1.2	−6.5
	(−441 to 215)	(−5.2 to 2.5)	(−27.8 to 13.5)

*Notes*: Table shows point estimates and 95% confidence intervals reflecting estimation uncertainty. Column 1 shows the estimated number of incremental doses delivered due to IMI. This result is calculated by generating predictions from regression models, with covariates set to their true values in the full set of IMI districts. Column 2 shows the estimated percentage point change in coverage in IMI districts. This result is calculated dividing the values in column 1 by an estimate of the target population. The target population is estimated using World Bank data on the population size, birth rate, and neonatal mortality rate in 2017, and HMIS data on the percentage of live births in India that occurred in IMI treatment districts. The full birth cohort is used as the target population for BCG, TT1, and TT2; the birth cohort size less the infant mortality rate is used as the target population for other infant vaccines. Column 3 shows the estimated percentage of unreached children in the treated districts who were reached through IMI. This result is generated by dividing the values in column 1 by an estimate of the number of children in the target districts who would have been unvaccinated if IMI had not occurred. The denominator is calculated by multiplying 1 minus the coverage in 2016 (from DHS estimates) by the size of the target population. NA indicates ‘Not Applicable.’

^a^ For these vaccines, there are no estimates of coverage in the 2016 DHS survey.

### Impact of IMI on coverage and the number of unvaccinated children

Based on these changes in vaccination volume, we estimated percentage point changes in coverage ranging from −1.2 (−5.2 to 2.5) for TT2 to 5.9 (−1.2 to 12.5) for DTP1, assessed over the full year following the beginning of implementation.

We estimated that IMI reduced the number of unvaccinated children by between 3.9% for HepB0 and 35.7% for DTP1, but these findings were not statistically significant. We estimated that IMI reduced the number of pregnant women missing TT1 by 0.7% (with a 95% CI ranging from a 47.2% decrease to a 49.9 increase), and increased the number of pregnant women missing TT2 by 6.5% (with a 95% CI ranging from a 27.8% decrease to a 13.5% increase).

### Effects of urbanization and baseline coverage

We found small and inconsistent results for the impact of urbanicity and baseline DTP3 coverage on IMI impact estimates. The estimated impact of IMI on coverage was higher in more urbanized areas for four of the vaccines evaluated (DTP1, OPV1, DTP3 and TT2), and lower for eleven vaccines. For baseline DTP3 coverage, the estimated impact of IMI was higher in districts with higher baseline coverage for nine of the vaccines evaluated, and lower for five vaccines. None of these differences were statistically significant. Detailed results are provided in Supplementary Table S2.

### Immunization sessions held and doses per session

**Figure 3:**
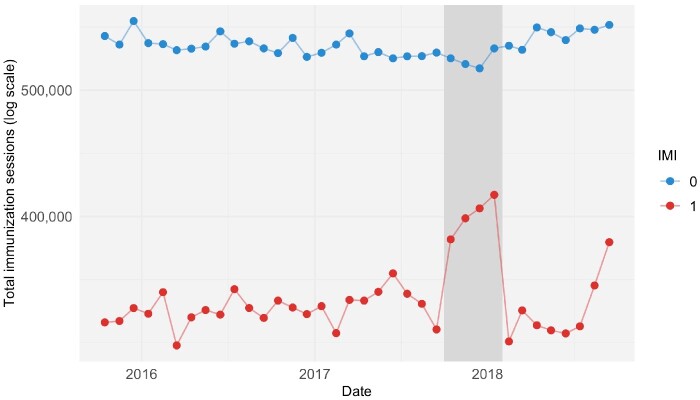
Increase in immunization sessions held during IMI implementation. *Notes*: This figure shows time trends in monthly total immunization sessions held in treated (red) and untreated (blue) districts. The dark grey bar indicates the period of IMI implementation, from October 2017 through January 2018. Raw counts were adjusted for seasonality by subtracting calendar month fixed effects estimated using linear regression models.

During implementation, IMI was estimated to have increased the number of immunization sessions held per treated district by 11.2% (95% CI 3.7 to 19.4) (Figure 3). For the two subsequent 4-month periods, the number of immunization sessions was estimated to be lower than in the absence of IMI, with treatment effects of −7.7% (95% CI −15.9 to 1.4) and −6.1% (95% CI −15.5 to 4.4), respectively. Assessed over the full year period, IMI was estimated to have produced limited changes in the total number of immunization sessions in the treated districts (decrease of 61 000, 95% CI −325 000 to 475 000).

During IMI implementation, the ratio of incremental infant vaccine doses to incremental immunization sessions held ranged from 0.1 (95% CI −0.9 to 0.9) doses of HepB0 per session to 2.9 (1.0 to 6.2) doses of DTP per session, and for maternal vaccines the ratio was −0.6 (−8.7 to 6.2) doses per session of TT1 and −0.2 (−1.3 to 0.8) doses of TT2.

### Sensitivity analyses

We found similar results across the eight alternative model specifications we tested, with some variation in point estimates (Supplementary Figure S6). The single interrupted time-series models generated similar point estimates (slightly higher than the main analysis for some vaccines and slightly lower for others), with narrower CIs than our main analysis for some vaccines. Notably, the point estimates from these models were approximately zero for the OPV0 and were negative for M1 (though the M1 estimates had very wide CIs). The analysis using coarsened exact matching generated similar results to the main analysis, again with higher estimates for some vaccines and lower estimates for others. Finally, when control districts that border on treatment districts were omitted from the analysis, the treatment effects were slightly higher but the CIs were much wider than in the main analysis. Detailed results from the sensitivity analyses are included in the Supplementary Tables S6–S12.

## Discussion

IMI represents one of the largest efforts to improve routine immunization coverage using campaign methods that has ever been attempted. The focus of IMI was to reach children and pregnant women previously unreached by the vaccination program, to increase coverage and improve equity. Evidence on the effectiveness of this approach can be used to guide future investments in similar types of approaches in India and elsewhere.

We found that IMI substantially increased delivery for 13 infant vaccines but not for 2 vaccines for pregnant women. During implementation, IMI increased vaccine delivery volume by between 1.6 (95% CI −6.4 to 10.2) and 13.8 (3.0 to 25.7)% across different vaccines, with a median effect of 10.6 (5.1 to 16.5). Assessed over a full year, IMI increased infant vaccine coverage by between 1.6 (−3.5 to 5.9) and 4.8 (−2.7 to 11.7) percentage points across different vaccines, reaching between 3.9% (−6.9 to 13.7) and 35.7% (−7.5 to 77.4) of children who otherwise would not have been reached, if the assumptions of the CITS are met. Among infant vaccines, the largest estimated effects were for DTP1 and OPV1, which suggest that IMI was able to reach previously unvaccinated (‘zero dose’) children. The smallest effects were for HepB0 and OPV0, and these effects were not statistically discernable from zero; this was expected because these vaccines are only administered up to 24 h and 15 days after birth, respectively, so children who missed these vaccines were not likely to be still eligible to receive them during IMI sessions. We found a negligible effect on the delivery of TT1 to pregnant women, and a slightly negative (though not statistically significant) effect on the delivery of TT2 to pregnant women.

Previous RCTs that evaluated interventions similar to IMI, involving immunization outreach at sites closer to communities and social mobilization to increase awareness of vaccination services, found larger effects on coverage (Oyo-Ita *et al.*, 2016; Banerjee *et al.*, 2010). However, interventions that are successful in the context of an RCT do not always have the same effect when implemented at scale. Outside of a trial setting, the intervention may not be as tailored to the population’s needs, and adherence to program design may not be as consistent. The smaller effect of IMI could also be due in part to low efficiency: while the number of immunization sessions significantly increased during IMI implementation, the efficiency of these sessions (measured as doses delivered per session) was low. We estimated that for infant vaccines, between 0.9 and 2.2 additional doses were delivered per additional session held during the implementation period. This could be because awareness or demand was low, or because the session sites were not optimally located. Further research would be needed to determine how to improve implementation efficiency, such as through incorporating text message reminders into social mobilization activities (Mekonnen *et al.*, 2019) or better targeting the session sites.

An earlier evaluation of IMI also estimated larger (though directionally similar) effects (Gurnani *et al.*, 2018). The earlier study used data from household surveys, which have several advantages over the HMIS data used in our study and can provide direct estimates of coverage. However, this prior study relied on baseline data from 2 years before the start of IMI and was not able to control for time trends in vaccine delivery that would have occurred in the absence of IMI. This approach would lead to overestimates of the impact of IMI if there were secular improvements in coverage over the 2-year period not attributable to IMI. In addition, the prior study did not assess the potential for a rebound effect, whereby vaccination delivery volume decreased in the months following IMI implementation. Our results suggest that evaluations of campaign-style interventions should include a sufficiently long post-period to account for the possibility of rebound after implementation.

While one of the objectives of the IMI program was to have a sustained effect (by raising awareness of the routine immunization program and improving routine planning), we did not find evidence for a sustained effect during the 8-months after IMI implementation ended. During the first 4 months after implementation, we found a small rebound effect: vaccination volume decreased, though not by a statistically significant amount. During the subsequent 4 months, vaccination volume increased again, though again not by a statistically significant amount.

The presence of a rebound directly after implementation suggests that, for some children, IMI may have improved vaccination timeliness. Reducing delays in immunization can improve health outcomes by to reducing exposed time, though this was not IMI’s objective (Clark and Sanderson, 2009). The lack of evidence for a sustained effect on coverage may be because IMI did not address long-term health system gaps, which include both supply-side issues such as human resource constraints and last-mile supply chain challenges, and demand-side issues such as low vaccine awareness and vaccine hesitancy. The reasons for lack of sustained impact need further study to inform the design of future interventions. In addition, there is a need to understand whether, and to what extent, this program diverted resources away from other routine health services, especially if policymakers are considering repeated implementation of IMI or similar efforts. When considering trade-offs between PIRI approaches and other approaches to improving coverage, such as investing in human resources, it is important to consider sustainability and the effects on other routine health services.

Although IMI had a special focus on reaching children in urban slum areas and in the poorest performing districts, we did not find substantial variation in treatment effect size by urbanization or baseline coverage (Ministry of Health & Family Welfare, Government of India, 2017). Differences in treatment effect size by urbanization and baseline coverage were mostly small and statistically insignificant. This could be driven by our use of district-level data, as it is possible that there was more meaningful variation by urbanization and coverage within districts. It is important to note that all districts included in IMI were selected for weak immunization performance, so, by increasing coverage in the treated districts, IMI had a meaningful impact on geographic equity in India even if the treatment effect size was similar across treated districts.

While IMI targeted pregnant women in addition to children, we estimated that it had a negligible or negative effect on the two maternal vaccines in our study. Baseline coverage of maternal vaccination was high relative to coverage of childhood vaccination, possibly due to the success of India’s cash transfer program for pregnant women, Janani Suraksha Yojana. IMI program implementation efforts were particularly focused on childhood vaccination. In addition, the maternal vaccination schedule is more specific than the childhood vaccination schedule (with shorter opportunities for catch-up).

We used a rigorous quasi-experimental approach to estimate the causal impact of IMI on vaccine delivery. Our approach accounted for secular changes in vaccine delivery volumes that would have occurred in the absence of IMI, for time invariant confounders, and for events or policies coinciding in time with IMI that would have affected vaccine delivery in both the treated and untreated districts. The use of high-frequency (monthly) data allowed us to estimate the effects of IMI both during implementation and afterwards to examine whether the size of the treatment effect changed over time.

Our study has several limitations. First, because HMIS data are noisy, our estimates have low precision. We used a quasi-Poisson distribution to accurately estimate uncertainty in the presence of over-dispersion in HMIS data. While our point estimates for the treatment effect of IMI over a 12-month period are large in magnitude, many of the estimates are statistically insignificant. Second, routinely collected data, such as HMIS data, can be subject to over- or under-reporting. Misreporting that was random, consistent over time or followed similar trends in treated and untreated districts would not bias our study findings. However, our findings could be biased if IMI influenced reporting practices. While we cannot know for sure, we do not believe this happened: IMI doses were reported into the HMIS system the same way as other doses, and reward payments for health workers did not change during IMI. Third, due to changes in administrative districts over time and omissions from the HMIS dataset, our sample omitted 8 treated and 91 untreated districts. The majority of the omitted untreated districts were from three states that had little to no IMI implementation, and their inclusion or exclusion in the control group would not be expected to change the results. The remaining omitted untreated districts and the omitted treated districts made up only a small portion of the overall birth cohort, so are also not expected to have a substantial effect on the results. Fourth, our main analysis used data on vaccination volume rather than direct measures of coverage. We therefore used auxiliary data sources to calculate target population size for estimating impacts on coverage and the percent reduction in the number of unvaccinated children. The accuracy of these impact estimates relies on the accuracy of the auxiliary data. Fifth, our causal inference approach relied on the untestable assumption that vaccine delivery trends would have changed the same way in treated and untreated districts if IMI had not occurred. We tested the robustness of our results to a wide range of model specifications and comparison groups and found qualitatively similar results. Sixth, there were changes in the delivery of the measles vaccine in India that coincided with the study period. As a result, our estimates of the impact of IMI on delivery of M1 and M2 may be less reliable than our estimates for other vaccines. Seventh, our analysis could be strengthened through the use of data on vaccine supply stock and flow, as collected in the electronic vaccine intelligence network (eVIN), but we did not have access to these data. Finally, we did not evaluate the impact of IMI on childhood vaccines delivered after 2 years, because the target population was under 2 years, but it is possible that IMI also had an impact on vaccine delivery to the older age group.

IMI was a major effort to improve immunization coverage in India. During the 3-month implementation period, we estimated substantial increases in coverage for multiple vaccines. However, these increases in coverage were not observed for all vaccines and were not always statistically significant. We estimated minimal impacts on maternal vaccination coverage and minimal impacts after the implementation period. PIRI may be an effective strategy for reaching zero dose children, increasing routine childhood immunization coverage, and improving equity, but may need to be followed-up with strong routine services to have long-lasting impact. Further work is needed to understand the sustainability and cost-effectiveness of this approach compared with other methods for increasing coverage, particularly for the unreached populations.

## Supplementary data

Supplementary data are available at *Health Policy and Planning* online.

## Supplementary Material

czab026_Supp
