# Supplementary material for: Impact of campaign-style delivery of routine vaccines: a quasi-experimental evaluation using routine health services data in India
Source: Health Policy Plan. 2021 Mar 18;36(4):454–63. doi: 10.1093/heapol/czab026 (PMC8128004; doi:10.1093/heapol/czab026)
Supplement: czab026_Supp [file czab026_supp.zip › czab026_Supplementary_Data.pdf]

SUPPLEMENTARY APPENDIX TO
Impact of Campaign-Style Delivery of Routine Vaccines: A Quasi-Experimental Evaluation using Routine Health Services Data in India

Appendix A: Statistical methods 2

Appendix B: Sensitivity analyses 3

Table S1: District sample size 5

Figure S1: Covariate balance before matching (dataset for Models 1 and 4) 6

Figure S2: Covariate balance after matching by region, DTP3 coverage, and MI participation (dataset for Model 5) 7

Figure S3: Covariate balance after matching by state, DTP3 coverage, and MI participation (dataset for Model 6) 8

Figure S4: Covariate balance after restricting the control group to non-contiguous districts (dataset for Model 7) 9

Figure S5: Covariate balance after restricting the control group to non-contiguous districts and matching by DTP3 coverage and MI participation (dataset for Model 8) 10

Table S2: Regression results: percent change in doses delivered in treated districts 11

Figure S6: Results from sensitivity analyses: percentage point change in annual coverage due to IMI 12

Table S3: Variation in impact of IMI on coverage (over a 1-year period) 14

Table S4: Variable names in regression output tables 15

Table S5: Results from Model 1 (controlled interrupted time-series using panel data with the full sample of treated and untreated districts) 16

Table S6: Results from Model 2 (single interrupted time-series using aggregate data) in the treated districts 17

Table S7: Results from Model 3 (single interrupted time-series using panel data from treated districts only) 18

Table S8: Results from Model 4 (single interrupted time-series using panel data from treated districts, with regression control for the target population size) 19

Table S9: Results from Model 5 (controlled interrupted time-series using a matched set of control districts; matching on region, coverage, and MI participation) 20

Table S10: Results from Model 6 (controlled interrupted time-series with matching by state, coverage, and MI participation) 21

Table S11: Results from Model 7 (controlled interrupted time-series using non-contiguous control districts) 22

Table S12: Results from Model 8 (controlled interrupted time-series using non-contiguous control districts matched on coverage and MI participation) 23

Appendix A: Statistical methods

Controlled interrupted time-series analysis: Single interrupted time-series studies model time trends in outcomes in the treated group, relying on the assumption that the outcome would have continued on their previous trend if treatment had not occurred [1]. Any deviation from the trend at the time of the treatment is assumed to be the result of the treatment. This method can be biased if an event coinciding in time with the treatment might have affected the outcome trend. Controlled interrupted time-series (CITS) models incorporate a comparison group to enable both before-after and between-group comparisons [2]. Controlled interrupted time-series studies rely on a weaker assumption than single interrupted time-series: these studies assume that, in the absence of treatment, any deviations from the trend in the treatment group coinciding with the time of the treatment would have been the same as the deviations in the control group (on the scale used in the model).

In our main analysis, we conducted a CITS analysis. The treatment group included all districts in the study sample that participated in IMI. The comparison group included all districts in the study sample that did not participate in IMI.

Regression models: Our main models took the form:

$$y_{it} \sim QuasiPoisson(\lambda_{it}, \delta)$$

$$\begin{aligned}\ln(\lambda_{it}) = {} & \alpha_{1-11} Month_t + \alpha_{12} Time_t + \alpha_{13} IMI_i * Time_t + \\ & \alpha_{14} Post1_t + \alpha_{15} Post2_t + \alpha_{16} Post3_t + \alpha_{17-(\sum i-1)} District_i + \\ & \beta_1 Post1_t * IMI_i + \beta_2 Post2_t * IMI_i + \beta_3 Post3_t * IMI_i + \\ & \beta_4 Post1_t * IMI_i * urban_i + \beta_5 Post2_t * IMI_i * urban_i + \beta_6 Post2_t * IMI_i * urban_i + \\ & \beta_7 Post1_t * IMI_i * cov_i + \beta_8 Post2_t * IMI_i * cov_i + \beta_9 Post2_t * IMI_i * cov_i\end{aligned}$$

where y_{ist} is the value of outcome y measured in district *i* at time *t*; λ_{it} is the modelled mean of y_{it}, δ is a dispersion factor; $Month_t$ is a set of 11 dummy variables for calendar months; $District_i$ is a set of district fixed effects; $Time$ is a continuous measure of time (in years); IMI_i is an indicator for whether district *i* was in the IMI program; $Post1_t$ is an indicator variable for whether an observation occurs from October 1, 2017 through January 31, 2018; $Post2_t$ is an indicator variable for whether an observation occurs from February 1, 2018 through May 31, 2018; and $Post3_t$ is an indicator variable or whether an observation occurs from June 1, 2018 through September 30, 2018; $urban_i$ is the estimated percent of children in district *i* living in urban areas in the 2016 DHS survey; and $coverage_i$ is the coverage of the DTP3 vaccine among eligible children in district *i* in the 2016 DHS survey.

Appendix B: Sensitivity analyses

We tested the robustness of our results to several different model specifications and different approaches to selecting a comparison group.

Models 2 and 3: Single interrupted time-series with pooled and panel data

First, to assess whether our results were influenced substantially by trend changes in the comparison group, we estimated single interrupted time-series models (as is recommended in comparative interrupted time-series studies [2]). We did this in two ways: first, using aggregated data with one observation per month (Model 2 in sensitivity analysis results) and then using panel data at the district-month level as in our main analysis (Model 3). By using an aggregated model, we also test the extent to which our results are driven by our use of district-level data (which gives more weight to small districts than aggregate data would). The aggregated models took the form:

$$y_{it} \sim QuasiPoisson(\lambda_{it}, \delta)$$

$$\ln(\lambda_{it}) = \alpha_{1-11} Month_t + \alpha_{12} Time_t + \beta_1 Post1_t + \beta_2 Post2_t + \beta_3 Post3_t$$

where the three beta coefficients represent the treatment effect estimate. The panel single interrupted time-series models, which include district-level covariates, took the form:

$$y_{it} \sim QuasiPoisson(\lambda_{it}, \delta)$$

$$\begin{aligned} \ln(\lambda_{it}) = {} & \alpha_{1-11} Month_t + \alpha_{12} Time_t + \\ & \beta_1 Post1_t + \beta_2 Post2_t + \beta_3 Post3_t + \\ & \beta_4 Post1_t * urban_i + \beta_5 Post2_t * urban_i + \beta_6 Post2_t * urban_i + \\ & \beta_7 Post1_t * cov_i + \beta_8 Post2_t * cov_i + \beta_9 Post2_t * cov_i \end{aligned}$$

where the nine beta coefficients represent the treatment effect estimate.

Model 4: Single interrupted time-series, controlling for target population size

Next, to control for potential time-varying confounding by target population size, we estimated single interrupted time-series models controlling for the number of children eligible for each vaccine (Model 4). These models used panel (district-month level) data. We estimated the number of eligible children by lagging data on live births from the HMIS system. For example, since the DTP1 vaccine is administered to infants at 6 weeks of age, we estimated the target population size for this vaccine by lagging live births by one month.

Models 5 and 6: Controlled interrupted time-series with matched comparison groups

Our CITS analysis could be biased if some event, coinciding in time with IMI, affected outcome trends in the treated and untreated districts differently. For example, this might occur if a weather event or policy change affected districts with certain characteristics, and the treated and untreated districts differed on those characteristics. To account for this possible situation, we conducted several matched analyses, analyzing outcome trends in a subset of treated and untreated districts with similar characteristics.

We used coarsened exact matching, which ensures multivariate balance on the variables included in the matching algorithm [3]. This approach resulted in a smaller sample that has different characteristics from the overall treatment group, but it has the advantage of improving the balance between treated and controlled districts (Figures S1-S5).

We first matched on region, DTP3 coverage at baseline, and participation in Mission Indradhanush (MI) (Model 5). We measured participation in MI as a categorical variable, with three categories: (1) participation in the most recent wave of MI; (2) participation in other waves of MI, but not the recent wave; and (3) no participation in MI.

We then matched on state, DTP3 coverage at baseline, and participation in MI (Model 6). Matching on state, rather than region, resulted in a smaller sample size but potentially better confounding control if confounding events occurred at the state level.

Models 7 and 8: Controlled interrupted time-series with non-contiguous control districts

Our CITS analysis could also be biased if there were spillover effects from IMI. For example, families in control districts neighboring on treated districts might have benefited from social mobilization activities in treated districts, or they might have brought their children to be vaccinated at immunization sessions held in treated districts. To account for this, we conducted a sensitivity analysis restricting the comparison group to districts that do not border treated districts (Model 7). We also conducted a sensitivity analysis restricting the comparison group to districts that do not border treated districts and matching (using coarsened exact matching) on baseline DTP3 coverage and participation in MI.

Table S1: District sample size

	Number of control districts *(live births in 2017)*	Number of treated districts *(live births in 2017)*	Total *(live births in 2017)*
Total in India in 2017	549	187	736
a) Full (raw) HMIS dataset	553 *(13,370,522)*	188 *(7,470,863)*	741 *(20,841,385)*
b) After correcting for name changes in the HMIS system	528 *(13,370,522)* *This indicates that 21 control districts are missing from the HMIS system.*	183 *(7,470,863)* *This indicates that 4 IMI districts are missing from the HMIS system.*	711 *(20,841,385)* *This indicates that 25 districts are missing from the HMIS system.*
c) After merging together districts that split at some point since 2012 (as long as both members of the split were in the same treatment group) in order to enable merge with DHS data[1]	513 *(13,370,522)*	179 *(7,470,863)*	692 *(20,841,385)*
d) After removing districts with significant administrative changes during the study period, which could not be resolved in Step (b).[2]	426 *(11,054,195)*	175 *(7,312,093)*	601 *(18,366,288)*
e) After removing districts with incomplete time-series data for two years prior to the start of the intervention[2]	424 *(11,032,573)*	175 *(7,312,093)*	599 *(18,344,666)*
f) Sample for Model 1	424 *(11,032,573)*	175 *(7,312,093)*	599 *(18,344,666)*
g) Sample for Models 2-4	0	175	175
h) Sample for Model 5	220 (*6,011,962*)	139 (*5,563,880*)	359 (*11,575,842*)
i) Sample for Model 6	107 (*3,688,734*)	91 (*3,196,589*)	198 (*6,885,323*)
j) Sample for Model 7	220 (*4,939,253*)	175 (*7,312,093*)	395 (*12,251,346*)
k) Sample for Model 8	219 (*4,937,477*)	130 (*5,145,002*)	349 (*10,082,479*)
l) Sample for Model 9	424 *(11,032,573)*	175 *(7,312,093)*	599 *(18,344,666)*

[1]The goal of this step was to enable merging of the HMIS dataset with India's Demographic and Health Surveys (DHS) data from 2016. India's 2016 DHS survey used district and state definitions 2012. In some cases, districts merged or split between 2012 and the study period (2017-2019). For example, Kra Daadi (AP) and Kurung Kumey (AP), both treatment districts, were part of the same district in 2012 but split in 2015. For the purposes of this analysis, we merged Kra Daadi and Kurung Kumey into one district by adding together the doses delivered in the two districts. This allowed us to merge the HMIS data with the DHS data, since the DHS survey only had data on the combined district.

[2] The goal of these two steps was to create a dataset that followed each district for two years prior to IMI implementation and one year after the start of IMI implementation. If administrative changes over time were too substantial to be resolved in step (c), then districts had to be omitted because they could not be followed over time.

Figure S1: Covariate balance before matching (dataset for Models 1 and 4)

A) Baseline DTP3 coverage

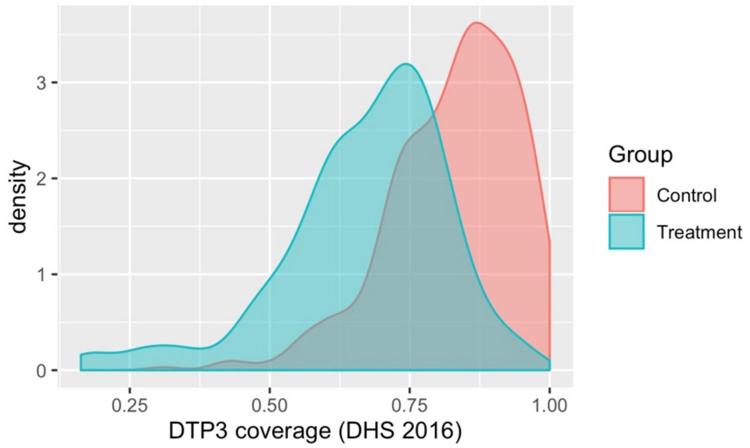

B) Participation in Mission Indradhanush

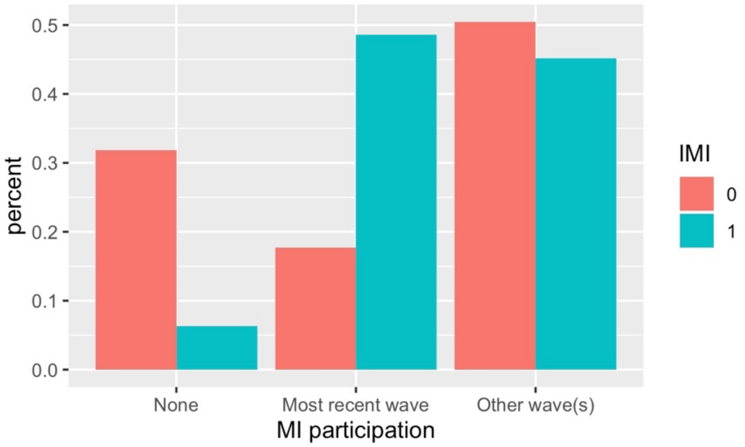

C) Percent of children whose mothers can read

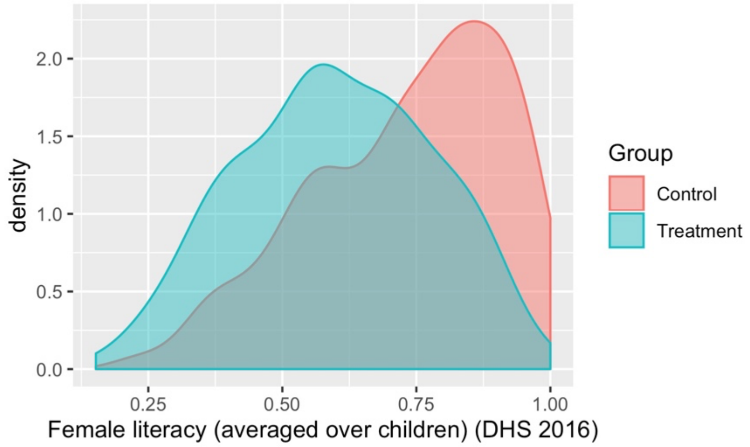

Notes: This figure shows covariate balance between treated (IMI) and control (non-IMI) districts in the full study sample, used for analysis in Models 1 and 4.

Figure S2: Covariate balance after matching by region, DTP3 coverage, and MI participation (dataset for Model 5)

A) Baseline DTP3 coverage

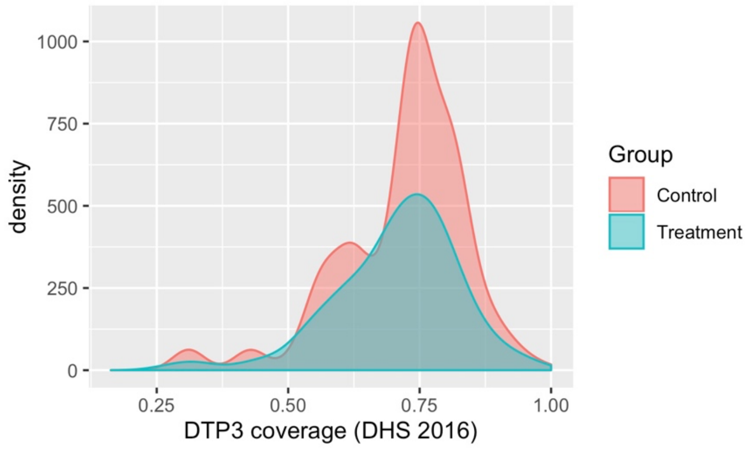

B) Participation in Mission Indradhanush

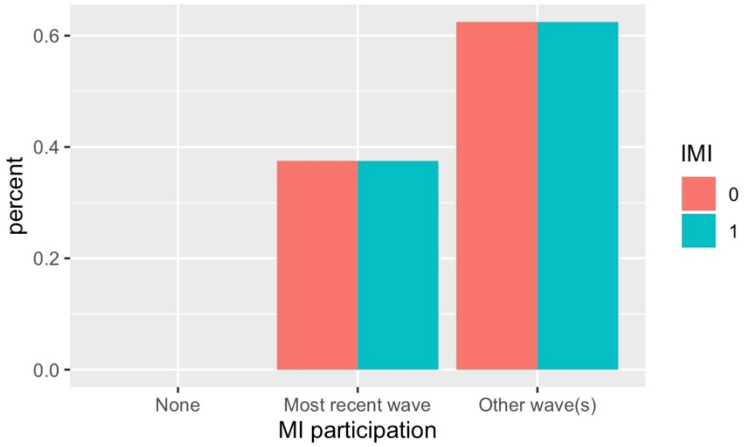

C) Percent of children whose mothers can read

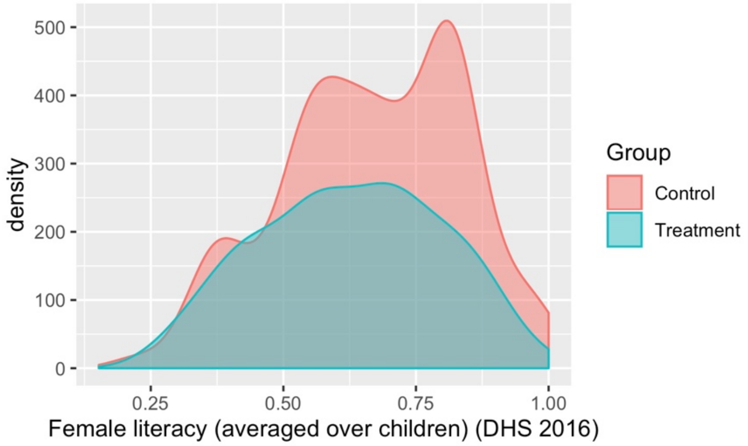

Notes: This figure shows covariate balance between treated (IMI) and control (non-IMI) districts in the matched study sample, used for analysis in Model 5. Weights from coarsened exact matching were applied before plots were generated.

Figure S3: Covariate balance after matching by state, DTP3 coverage, and MI participation (dataset for Model 6)

A) Baseline DTP3 coverage

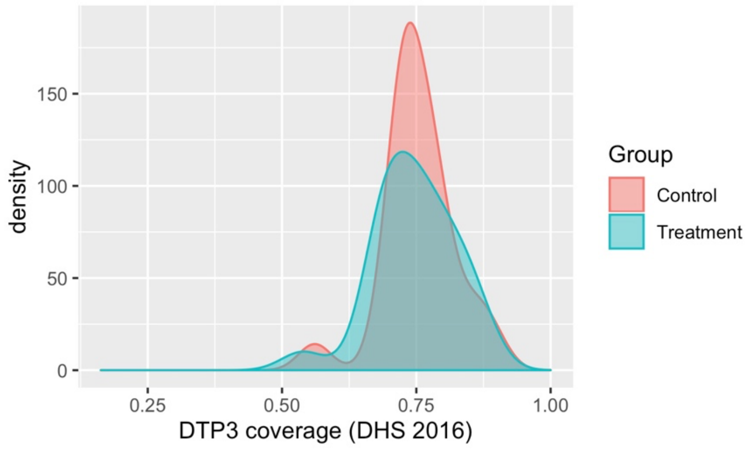

B) Participation in Mission Indradhanush

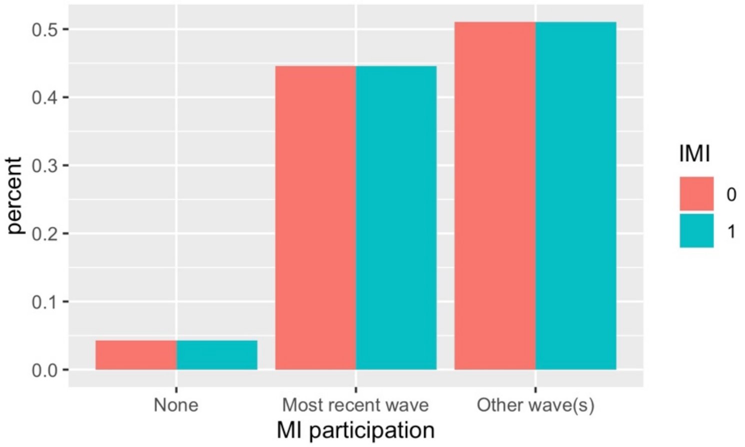

C) Percent of children whose mothers can read

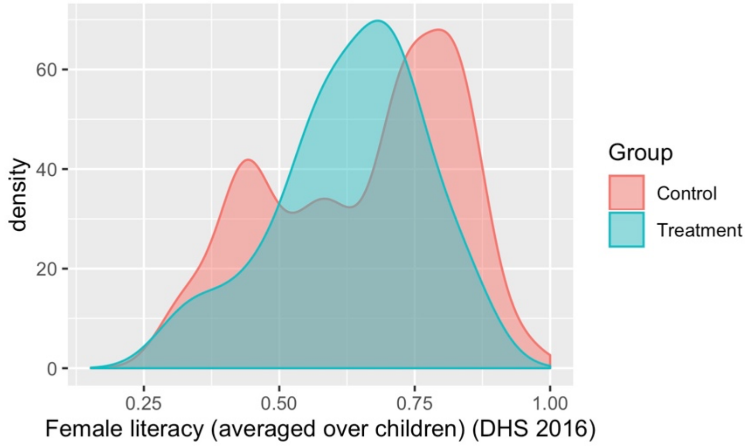

Notes: This figure shows covariate balance between treated (IMI) and control (non-IMI) districts in the matched study sample, used for analysis in Model 6. Weights from coarsened exact matching were applied before plots were generated.

Figure S4: Covariate balance after restricting the control group to non-contiguous districts (dataset for Model 7)

A) Baseline DTP3 coverage

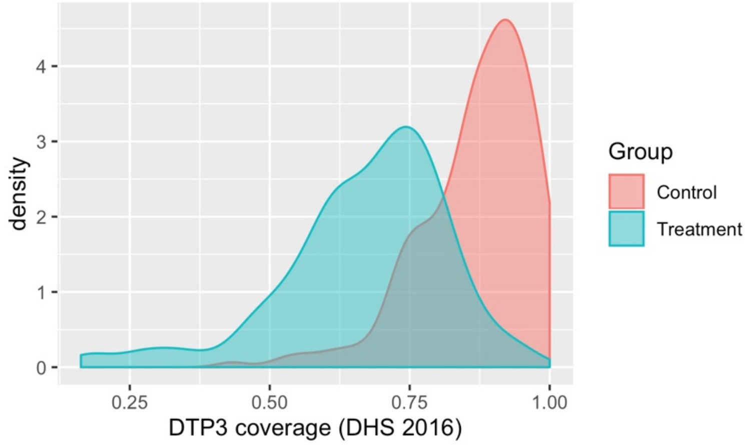

B) Participation in Mission Indradhanush

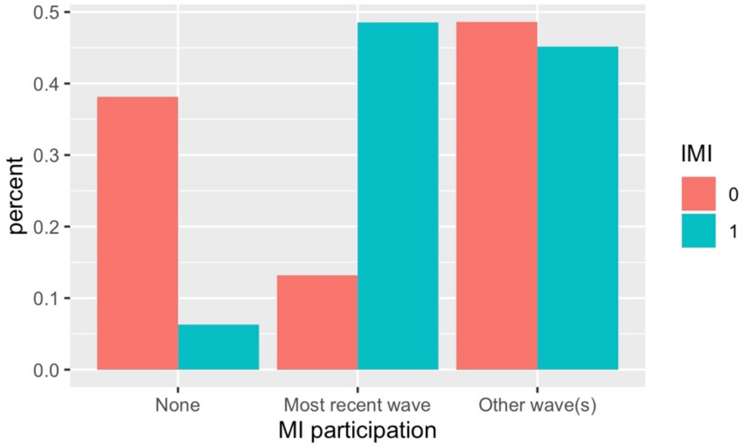

C) Percent of children whose mothers can read

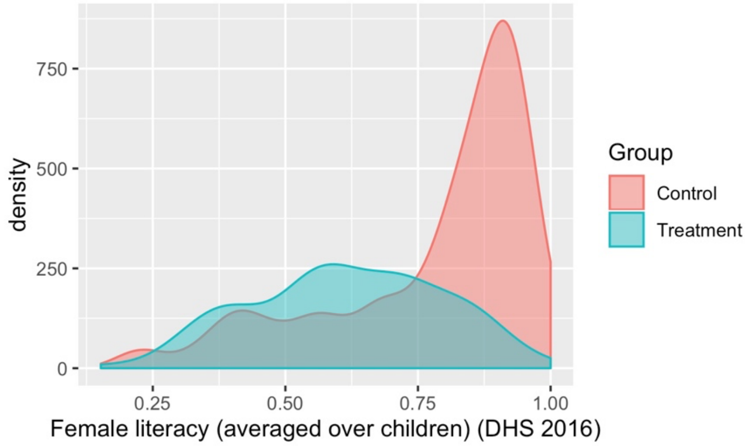

Notes: This figure shows covariate balance between treated (IMI) and control (non-IMI) districts in the study sample, with the control group restricted to districts that did not border treatment districts (as used for analysis in Model 7).

Figure S5: Covariate balance after restricting the control group to non-contiguous districts and matching by DTP3 coverage and MI participation (dataset for Model 8)

A) Baseline DTP3 coverage

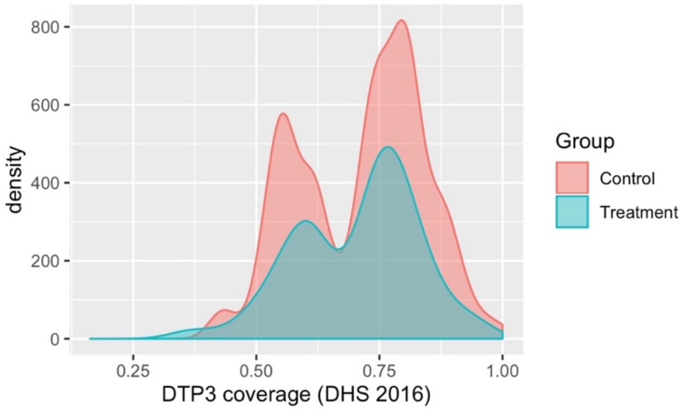

B) Participation in Mission Indradhanush

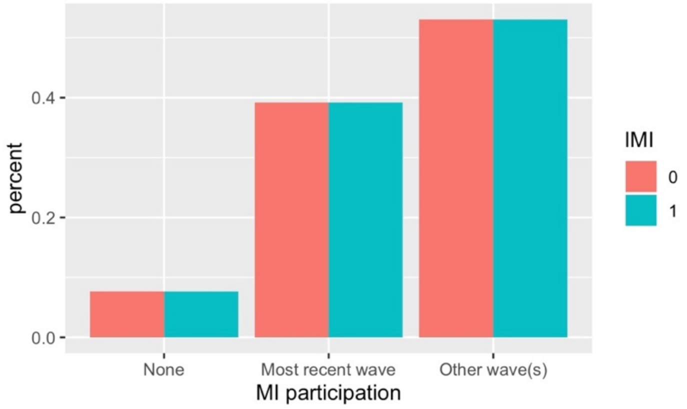

C) Percent of children whose mothers can read

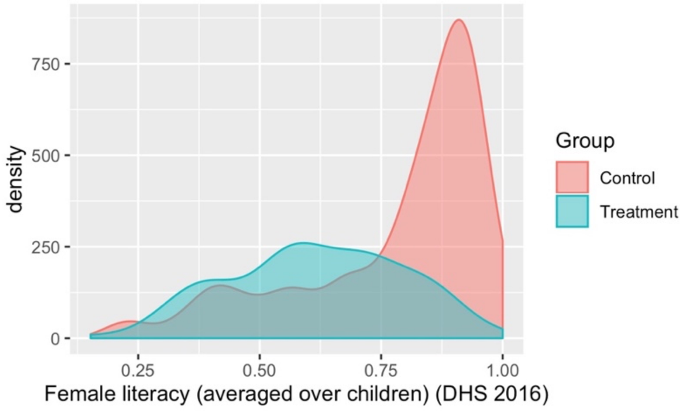

Notes: This figure shows covariate balance between treated (IMI) and control (non-IMI) districts in the sample used for analysis in Model 8. The control group was restricted to untreated districts that did not border treated districts, and then coarsened exact matching was used. Weights from coarsened exact matching were applied before plots were generated.

Table S2: Regression results: percent change in doses delivered in treated districts

Vaccine	(1) Months 1-4 (during IMI implementation)	(2) Months 5-8 (immediately after implementation ended)	(3) Months 9-12 (four months after implementation ended)
BCG	9.0<br>(2.8, 15.6)	-1.8<br>(-9.4, 6.3)	4.1<br>(-3.8, 12.6)
HepB0	1.6<br>(-6.4, 10.2)	1.7<br>(-9.6, 14.3)	10.1<br>(-2.5, 24.4)
OPV0	3.9<br>(-2.1, 10.3)	0.0<br>(-8.7, 9.5)	5.9<br>(-3.1, 15.8)
DTP1	12.8<br>(5.3, 21.0)	0.9<br>(-6.0, 8.2)	3.6<br>(-4.2, 11.9)
OPV1	11.9<br>(3.8, 20.7)	0.3<br>(-7.1, 8.3)	2.3<br>(-6, 11.3)
DTP2	10.6<br>(5.1, 16.5)	-0.7<br>(-7.5, 6.6)	2.7<br>(-5.0, 11.1)
OPV2	10.3<br>(4.6, 16.4)	-0.9<br>(-7.8, 6.5)	1.9<br>(-5.8, 10.4)
DTP3	10.0<br>(4.4, 15.8)	-2.1<br>(-8.6, 4.8)	2.3<br>(-4.9, 10.0)
OPV3	9.6<br>(4.1, 15.4)	-2.8<br>(-9.1, 4.1)	1.4<br>(-5.6, 8.9)
M1	11.8<br>(8.2, 15.6)	-1.1<br>(-5.6, 3.7)	2.8<br>(-2.3, 8.3)
DTPb	13.8<br>(3.0, 25.7)	-4.4<br>(-11.6, 3.4)	1.1<br>(-7.6, 10.5)
M2	10.7<br>(1.8, 20.4)	-1.8<br>(-9.6, 6.6)	1.8<br>(-7.2, 11.8)
OPVb	12.3<br>(0.9, 24.9)	-6.4<br>(-14.2, 2.1)	-1.2<br>(-10.5, 9.1)
TT1	-1.7<br>(-8.0, 4.9)	-2.7<br>(-8.2, 3.0)	5.0<br>(-1.2, 11.5)
TT2	-0.2<br>(-4.8, 4.6)	-6.3<br>(-10.0, -2.5)	3.1<br>(-1.3, 7.7)

Notes: This table shows estimates of the effect of IMI on doses delivered, measured as a percent change in doses delivered in each of three time periods ((1) during implementation; (2) the four months following implementation; and (3) the four months after that. Covariate values (urbanization and baseline coverage) are fixed at their mean values. To show percent change, we first exponentiate regression coefficients (because models are fit with a log link function), then subtract one and multiply by 100. To calculate 95% confidence intervals on the same scale, we first calculate 95% confidence intervals on the log scale using assumptions from the normal distribution, and then exponentiate the interval bounds, subtract one, and multiply by 100.

Figure S6: Results from sensitivity analyses: percentage point change in annual coverage due to IMI

Panel A: Implementation period (Months 1-4)

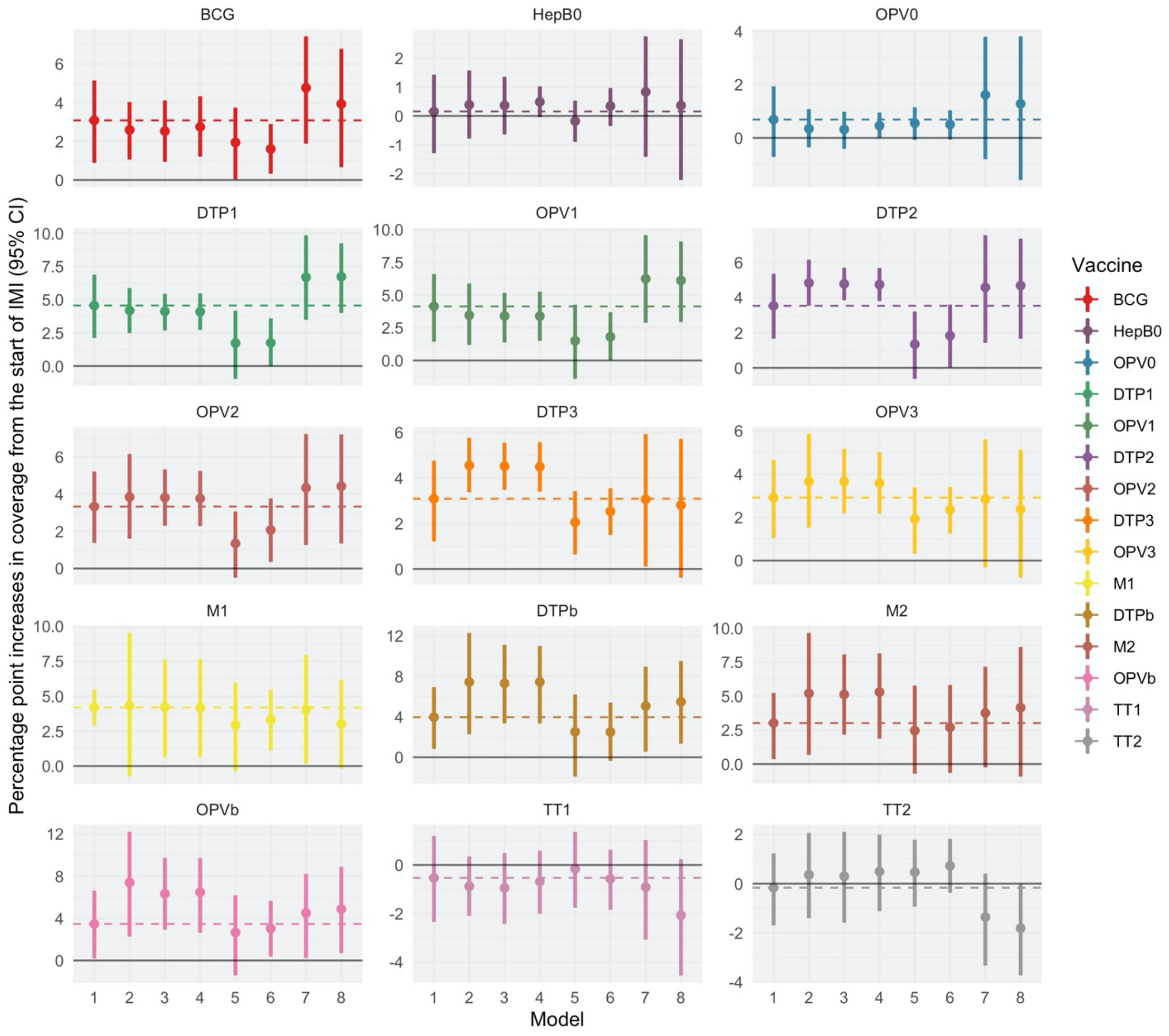

Panel B: Months 1-12

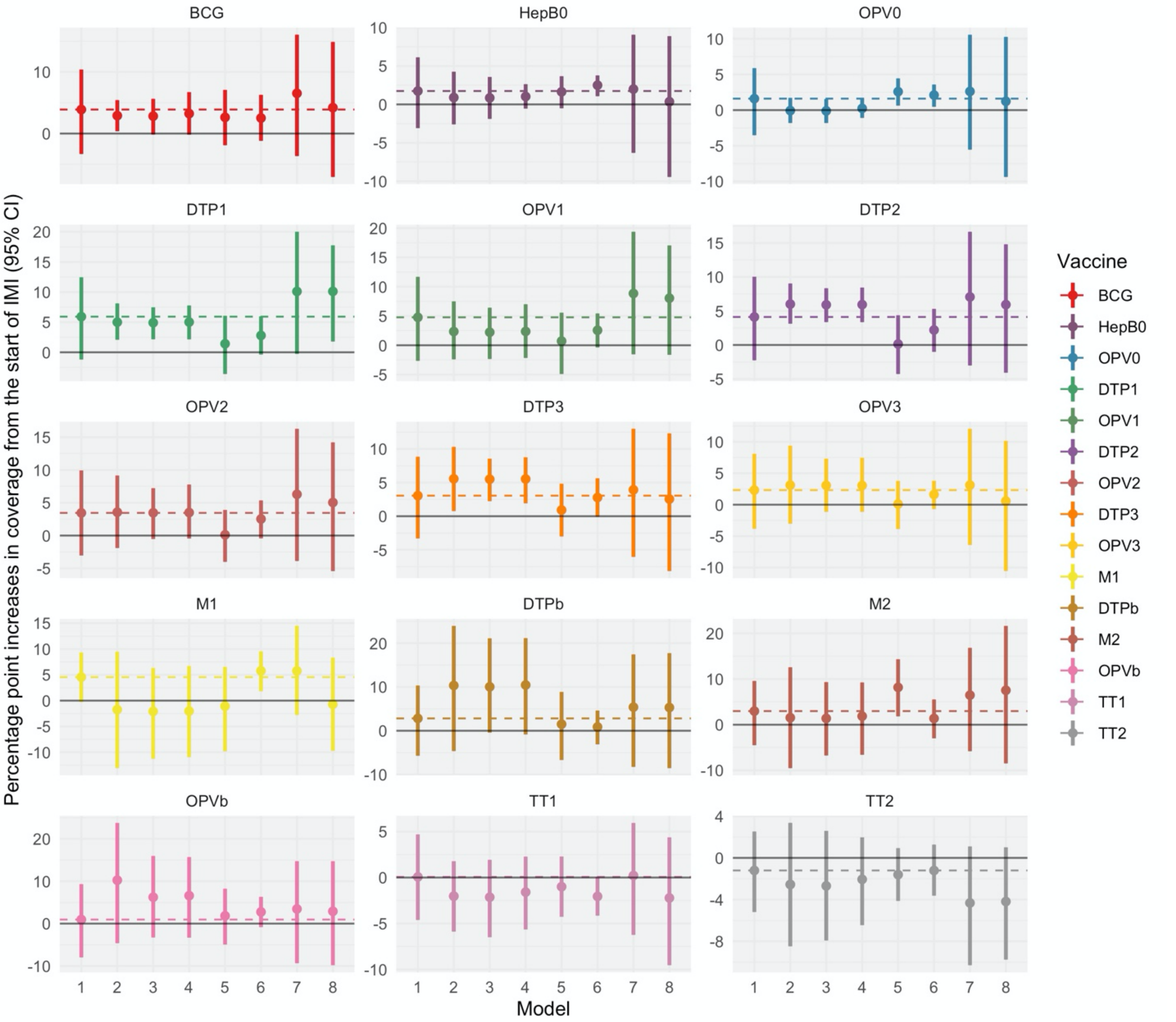

Notes: This figure shows point estimates and 95% confidence intervals for the effect of IMI on coverage. The first set of panels shows results for the implementation period and the second set of panels shows results for the full year from the start of implementation. The horizontal dotted line on each panel shows the point estimate from the Model 1, the main model. Model 1 is a comparative interrupted time-series, using all untreated districts in the study sample. Model 2 is a single interrupted time-series, with all data from the treated districts pooled into one observation per time period. Model 3 is a single interrupted time-series, with a panel data structure. Model 4 is a single interrupted time-series with a panel data structure, using regression control for potential time-varying confounding by target population size. Model 5 is a controlled interrupted time-series, with a control group selected using coarsened exact matching by region, baseline coverage, and MI participation. Model 6 is a controlled interrupted time-series, with a control group selected using coarsened exact matching by state, baseline coverage, and MI participation. Model 7 is a controlled interrupted time-series, with the comparison group consisting of all untreated districts that do not neighbor the treated districts. Finally, Model 8 is a controlled interrupted time-series, with a comparison group consisting of non-contiguous control districts that were matched to treated districts based on coverage and MI participation.

Table S3: Variation in impact of IMI on coverage (over a 1-year period)

	**Variation by baseline DTP3 coverage**	**Variation by urbanization**
	Treatment effect in districts with 76% baseline DTP3 coverage minus treatment effect in districts with 60% baseline coverage (95% CI)	***Treatment effect in districts with 28.4% urbanization minus treatment effect in districts with 8.5% urbanization (95% CI)***
BCG	0.9<br>(0.1, 1.7)	-1.2<br>(-2.8, 0.3)
HepB0	-0.8<br>(-1.5, -0.2)	-1.4<br>(-2.5, -0.3)
OPV0	-0.2<br>(-0.8, 0.4)	-1.9<br>(-3.3, -0.6)
DTP1	0.7<br>(0.0, 1.5)	0.5<br>(-1.2, 2.1)
OPV1	0.2<br>(-0.6, 1.0)	0.2<br>(-1.6, 2.0)
DTP2	0.6<br>(-0.3, 1.5)	-0.3<br>(-1.6, 1.0)
OPV2	0.3<br>(-0.5, 1.2)	-0.5<br>(-1.9, 1.0)
DTP3	-0.2<br>(-1.0, 0.7)	0.1<br>(-1.0, 1.2)
OPV3	0.2<br>(-0.7, 1.2)	-0.3<br>(-1.6, 0.9)
M1	-0.7<br>(-1.9, 0.6)	-0.8<br>(-2.8, 1.4)
DTPb	0.3<br>(-0.7, 1.2)	-1.1<br>(-2.9, -0.4)
M2	1.2<br>(0.3, 2.2)	-0.8<br>(-2.7, 1.1)
OPVb	0.1<br>(-0.9, 0.9)	-0.8<br>(-2.1, -0.1)
TT1	-0.4<br>(-0.8, 0.1)	-0.2<br>(-1.3, 1.0)
TT2	1.6<br>(0.8, 2.5)	0.1<br>(-1.3, 1.5)

Notes: Column 1 shows how the estimated impact of IMI on vaccine coverage varies for districts with different baseline DTP3 coverage levels. For example, 76% baseline DTP3 coverage (equivalent to the 75th percentile among the treated districts) IMI has a 0.6 percentage point larger impact on coverage of DTP2 among districts with 76% baseline DTP3 coverage (equivalent to the 75th percentile among the treated districts) than among districts with 60% DTP3 coverage (equivalent to the 25th percentile among the treated districts. Equal-tailed 95% credible intervals are shown for results

Table S4: Legend for regression output tables

Variable name or abbreviation	Meaning
Date_inyears	Continuous variable measuring time in years
Post1	Dummy variable for the IMI implementation period (October 2017 through January 2018)
Post2	Dummy variable for the four months immediately following IMI implementation (February 2018 through May 2018)
Post3	Dummy variable for the four months from June 2018 through August 2018
IMI	Dummy variable for participation in IMI
Coverage	Baseline DTP3 coverage at the district level (measured using data from the 2016 DHS survey)
Urbanization	Percent of the district population living in an urban area (measured using data from the 2016 DHS survey)
Target pop	Target population size for each vaccine. This is estimated as the number of lagged live births, with the length of the lag depending on the outcome variable. The lag for BCG is 0 months, for DTP1 is 1 month, for DTP2 is 2 months, for DTP3 is 3 months, for M1 is 9 months, for M2 is 9 months, and for TT1 and TT2 is 0 months.
Sessions	Number of immunization sessions held per district-month
*	Statistically significant at the 0.10 level
**	Statistically significant at the 0.05 level
***	Statistically significant at the 0.01 level
se	Standard error

Table S5: Results from Model 1 (controlled interrupted time-series using panel data with the full sample of treated and untreated districts)

	BCG	HepB0	OPV0	DTP1	OPV1	DTP2	OPV2	DTP3	OPV3	M1	DTPb	M2	OPVb	TT1	TT2	Sessions
date_inyears	-0.014	0.055***	0.01	-0.001	0.011	-0.009	0.004	-0.009	0.002	0.067***	-0.039	0.002	-0.028	0.015	0.036***	-0.019**
s.e.	(0.010)	-0.014	-0.01	(0.008)	-0.009	(0.008)	-0.009	(0.009)	-0.009	(0.016)	-0.034	(0.024)	-0.025	(0.009)	(0.011)	(0.009)
post1	0.010	0.015	-0.002	0.016	0.009	0.034***	0.020*	0.027**	0.014	0.019	0.091	0.053	0.072*	-0.026	0.002	0.013
	(0.016)	-0.027	-0.019	(0.015)	-0.017	(0.012)	-0.011	(0.012)	-0.012	(0.034)	-0.058	(0.042)	-0.043	(0.017)	(0.019)	(0.013)
post2	-0.003	-0.019	-0.023	-0.001	-0.007	0.008	0.004	0.016	0.013	-0.055*	0.047	-0.048	0.04	-0.041***	-0.024	0.033*
	(0.024)	-0.028	-0.023	(0.016)	-0.015	(0.016)	-0.014	(0.016)	-0.016	(0.032)	-0.06	(0.037)	-0.041	(0.016)	(0.017)	(0.017)
post3	0.010	-0.062**	-0.042*	0.013	-0.02	0.021	-0.009	0.017	-0.009	-0.086***	0.095	-0.065	0.049	-0.008	-0.024	0.054**
	(0.023)	-0.028	-0.022	(0.019)	-0.021	(0.017)	-0.019	(0.017)	-0.018	(0.028)	-0.078	(0.059)	-0.059	(0.014)	(0.017)	(0.021)
IMI*post1	0.086***	0.016	0.038	0.121***	0.113***	0.101***	0.098***	0.095***	0.092***	0.112***	0.129**	0.102**	0.116**	-0.018	-0.002	0.107***
	(0.030)	-0.042	-0.03	(0.035)	-0.038	(0.026)	-0.027	(0.026)	-0.026	(0.017)	-0.051	(0.043)	-0.054	(0.033)	(0.024)	(0.036)
IMI*post2	-0.018	0.016	0	0.009	0.003	-0.007	-0.009	-0.022	-0.028	-0.011	-0.045	-0.018	-0.066	-0.028	-0.065***	-0.080*
	(0.041)	-0.06	-0.046	(0.036)	-0.039	(0.036)	-0.037	(0.035)	-0.035	(0.024)	-0.04	(0.042)	-0.044	(0.029)	(0.020)	(0.048)
IMI*post3	0.040	0.096	0.057	0.035	0.022	0.027	0.019	0.023	0.014	0.028	0.01	0.018	-0.012	0.049	0.031	-0.063
	(0.040)	-0.062	-0.045	(0.040)	-0.043	(0.040)	-0.04	(0.037)	-0.036	(0.026)	-0.046	(0.048)	-0.051	(0.031)	(0.022)	(0.054)
date_inyears*IMI	-0.022	-0.016	0	-0.026	-0.019	-0.015	-0.009	-0.004	0.004	0.018	0.019	0.026	0.03	-0.034**	0.006	0.040
	(0.016)	-0.028	-0.02	(0.016)	-0.018	(0.018)	-0.018	(0.018)	-0.016	(0.014)	-0.022	(0.020)	-0.023	(0.015)	(0.009)	(0.025)
IMI*post1*urban	-0.156***	-0.174***	-0.245***	-0.075	-0.097**	-0.041	-0.067*	-0.003	-0.026	-0.188***	-0.129***	-0.005	-0.106***	0.048	0.017	-0.116***
	(0.036)	-0.047	-0.044	(0.048)	-0.049	(0.038)	-0.038	(0.041)	-0.042	(0.045)	-0.047	(0.057)	-0.04	(0.056)	(0.050)	(0.029)
IMI*post2*urban	0.024	-0.03	-0.069	0.044	0.019	-0.032	-0.052	-0.015	-0.054*	0.013	-0.097***	-0.180***	-0.082***	0.031	0.092*	0.160***
	(0.049)	-0.082	-0.086	(0.034)	-0.039	(0.035)	-0.035	(0.028)	-0.029	(0.056)	-0.031	(0.069)	-0.027	(0.039)	(0.055)	(0.020)
IMI*post3*urban	-0.040	-0.156	-0.139*	0.094**	0.081	0.006	0.003	-0.029	-0.041	-0.002	-0.114**	-0.139	-0.088*	-0.061**	-0.102***	0.157***
	(0.052)	-0.095	-0.08	(0.041)	-0.054	(0.045)	-0.053	(0.037)	-0.05	(0.072)	-0.051	(0.093)	-0.052	(0.030)	(0.031)	(0.019)
IMI*post1*coverage	-0.137***	-0.014	-0.036	-0.246***	-0.280***	-0.336***	-0.371***	-0.445***	-0.448***	-0.432***	-0.261**	-0.335***	-0.335***	-0.201***	-0.196***	-0.105**
	(0.056)	-0.063	-0.044	(0.089)	-0.087	(0.075)	-0.069	(0.045)	-0.043	(0.045)	-0.109	(0.118)	-0.113	(0.047)	(0.042)	(0.041)
IMI*post2*coverage	0.093**	-0.125	-0.072	0.109**	0.082*	0.201***	0.161***	0.118*	0.142**	-0.173***	0.279***	0.323***	0.248***	0.079***	0.199***	-0.008
	(0.045)	-0.078	-0.065	(0.045)	-0.045	(0.051)	-0.046	(0.071)	-0.06	(0.044)	-0.055	(0.073)	-0.052	(0.028)	(0.027)	(0.043)
IMI*post3*coverage	-0.075	-0.214***	-0.214***	-0.181***	-0.218***	-0.154**	-0.197***	-0.122*	-0.123*	-0.385***	-0.171***	-0.173**	-0.178***	-0.068***	-0.037	-0.272
	(0.042)	-0.065	-0.049	(0.055)	-0.057	(0.069)	-0.06	(0.072)	-0.067	(0.054)	-0.065	(0.080)	-0.061	(0.024)	(0.033)	(0.051)
Observations	21,564	21,564	21,564	21,564	21,564	21,564	21,564	21,564	21,564	21,564	21,564	21,564	21,564	21,564	21,533	21,537

Table S6: Results from Model 2 (single interrupted time-series using aggregate data) in the treated districts

	BCG	HepB0	OPV0	DTP1	OPV1	DTP2	OPV2	DTP3	OPV3	M1	DTPb	M2	OPVb	TT1	TT2	Sessions
date	-0.025***	0.037	0.013	-0.013	0.006	-0.021*	-0.001	-0.016	0.005	0.091***	-0.025	0.026	-0.002	-0.02	0.040**	0.015
	-0.005	-0.023	-0.009	-0.008	-0.016	-0.011	-0.018	-0.014	-0.021	-0.024	-0.057	-0.035	-0.047	-0.014	-0.019	(0.012)
post1	0.066***	0.021	0.014	0.102***	0.084***	0.128***	0.101***	0.127***	0.102***	0.1	0.235***	0.166**	0.201**	-0.032	0.01	0.147***
	-0.021	-0.035	-0.015	-0.022	-0.031	-0.019	-0.031	-0.018	-0.032	-0.064	-0.091	-0.079	-0.082	-0.021	-0.026	(0.023)
post2	-0.040***	0.008	-0.018	-0.022	-0.032	-0.016	-0.019	-0.012	-0.021	-0.075*	-0.004	-0.08	-0.038	-0.078**	-0.085***	-0.049**
	-0.012	-0.033	-0.016	-0.014	-0.02	-0.017	-0.031	-0.022	-0.036	-0.045	-0.084	-0.054	-0.069	-0.033	-0.025	(0.022)
post3	0.045***	0.023	-0.001	0.048***	-0.001	0.053***	0.012	0.044	0.005	-0.072	0.108	-0.043	0.041	0.041*	0.005	-0.009
	-0.011	-0.045	-0.018	-0.013	-0.023	-0.019	-0.025	-0.045	-0.03	-0.047	-0.089	-0.064	-0.074	-0.024	-0.041	(0.044)
constant	13.540***	12.787***	13.100***	13.593***	13.583***	13.572***	13.583***	13.550***	13.555***	13.584***	13.319***	13.337***	13.345***	13.354***	13.475***	12.768***
	-0.016	-0.034	-0.017	-0.02	-0.024	-0.021	-0.029	-0.02	-0.028	-0.044	-0.088	-0.067	-0.077	-0.023	-0.024	(0.020)

Table S7: Results from Model 3 (single interrupted time-series using panel data from treated districts only)

	BCG	HepB0	OPV0	DTP1	OPV1	DTP2	OPV2	DTP3	OPV3	M1	DTPb	M2	OPVb	TT1	TT2	Sessions
date	-0.025***	0.037**	0.013	-0.013*	0.006	-0.021***	-0.001	-0.016	0.005	0.091***	-0.025	0.026	-0.002	-0.02	0.040***	0.015
	-0.006	-0.018	-0.008	-0.007	-0.013	-0.008	-0.012	-0.01	-0.014	-0.023	-0.038	-0.025	-0.031	-0.015	-0.015	(0.011)
post1	0.072***	0.027	0.023	0.108***	0.092***	0.136***	0.110***	0.137***	0.113***	0.115**	0.246***	0.175***	0.212***	-0.03	0.013	0.149***
	-0.022	-0.031	-0.016	-0.02	-0.026	-0.013	-0.021	-0.015	-0.021	-0.046	-0.069	-0.055	-0.061	-0.027	-0.029	(0.020)
post2	-0.043***	0.013	-0.014	-0.026*	-0.035	-0.02	-0.022	-0.014	-0.023	-0.071*	-0.009	-0.086*	-0.043	-0.080**	-0.092***	-0.050**
	-0.014	-0.027	-0.016	-0.014	-0.024	-0.013	-0.023	-0.017	-0.026	-0.042	-0.065	-0.044	-0.054	-0.031	-0.026	(0.020)
post3	0.047***	0.033	0.009	0.049***	0.002	0.056***	0.016	0.047*	0.009	-0.063*	0.115	-0.035	0.049	0.044*	0.008	-0.004
	-0.015	-0.037	-0.017	-0.014	-0.02	-0.019	-0.022	-0.025	-0.025	-0.038	-0.073	-0.049	-0.058	-0.027	-0.03	(0.040)
post1*urban	-0.156***	-0.174***	-0.245***	-0.075	-0.097**	-0.041	-0.067*	-0.003	-0.026	-0.188***	-0.129***	-0.005	-0.106***	0.048	0.017	-0.116***
	-0.036	-0.047	-0.044	-0.048	-0.049	-0.038	-0.038	-0.041	-0.042	-0.045	-0.047	-0.058	-0.04	-0.056	-0.05	(0.029)
post2*urban	0.024	-0.03	-0.069	0.044	0.019	-0.032	-0.052	-0.015	-0.054*	0.013	-0.097***	-0.180***	-0.082***	0.031	0.092*	0.160***
	-0.049	-0.082	-0.086	-0.034	-0.039	-0.035	-0.035	-0.028	-0.029	-0.056	-0.031	-0.069	-0.027	-0.039	-0.055	(0.020)
post3*urban	-0.04	-0.156	-0.139*	0.094**	0.081	0.006	0.003	-0.029	-0.041	-0.002	-0.114**	-0.139	-0.088*	-0.061**	-0.102***	0.157***
	-0.052	-0.095	-0.08	-0.041	-0.054	-0.045	-0.053	-0.037	-0.05	-0.072	-0.051	-0.093	-0.052	-0.03	-0.031	(0.019)
post1*coverage	-0.137**	-0.014	-0.036	-0.246***	-0.280***	-0.336***	-0.371***	-0.445***	-0.448***	-0.432***	-0.261**	-0.335***	-0.335***	-0.201***	-0.196***	-0.105**
	-0.056	-0.063	-0.044	-0.089	-0.087	-0.075	-0.069	-0.045	-0.043	-0.045	-0.109	-0.118	-0.113	-0.047	-0.042	(0.041)
post2*coverage	0.093**	-0.125	-0.072	0.109**	0.082*	0.201***	0.161***	0.118*	0.142**	-0.173***	0.279***	0.323***	0.248***	0.079***	0.199***	-0.008
	-0.045	-0.078	-0.065	-0.045	-0.045	-0.051	-0.046	-0.071	-0.06	-0.044	-0.055	-0.073	-0.052	-0.028	-0.027	(0.043)
post3*coverage	-0.075*	-0.214***	-0.214***	-0.181***	-0.218***	-0.154**	-0.197***	-0.122*	-0.123*	-0.385***	-0.171***	-0.173**	-0.178***	-0.068***	-0.037	-0.272***
	-0.042	-0.065	-0.049	-0.055	-0.057	-0.069	-0.06	-0.072	-0.067	-0.054	-0.065	-0.08	-0.061	-0.024	-0.033	(0.051)
Observations	6,300	6,300	6,300	6,300	6,300	6,300	6,300	6,300	6,300	6,300	6,300	6,300	6,300	6,300	6,300	6,299

Table S8: Results from Model 4 (single interrupted time-series using panel data from treated districts, with regression control for the target population size)

	BCG	HepB0	OPV0	DTP1	OPV1	DTP2	OPV2	DTP3	OPV3	M1	DTPb	M2	OPVb	TT1	TT2	Sessions
date_inyears	-0.019**	0.047***	0.021***	-0.011	0.008	-0.019**	0	-0.015	0.007	0.091***	-0.026	0.025	-0.003	-0.016	0.042***	0.016
	-0.009	-0.011	-0.005	-0.007	-0.013	-0.008	-0.013	-0.011	-0.014	-0.023	-0.038	-0.025	-0.031	-0.014	-0.013	(0.011)
post1	0.077***	0.037**	0.030***	0.107***	0.091***	0.134***	0.109***	0.135***	0.110***	0.115**	0.247***	0.177***	0.214***	-0.022	0.019	0.150***
	-0.023	-0.018	-0.011	-0.02	-0.026	-0.014	-0.021	-0.016	-0.022	-0.046	-0.07	-0.055	-0.061	-0.024	-0.024	(0.020)
post2	-0.037**	0.019	-0.006	-0.024	-0.033	-0.019	-0.021	-0.014	-0.023	-0.071*	-0.007	-0.084*	-0.041	-0.073**	-0.085***	-0.049**
	-0.017	-0.018	-0.014	-0.015	-0.024	-0.014	-0.024	-0.018	-0.027	-0.043	-0.065	-0.044	-0.054	-0.031	-0.023	(0.020)
post3	0.048***	0.033	0.012	0.050***	0.003	0.057***	0.016	0.047*	0.009	-0.063*	0.117	-0.034	0.05	0.046*	0.012	-0.004
	-0.015	-0.023	-0.01	-0.014	-0.021	-0.019	-0.023	-0.025	-0.025	-0.038	-0.073	-0.049	-0.057	-0.026	-0.026	(0.039)
Lagged births (1000s)	0.042***	0.072***	0.060***	0.011***	0.011**	0.008**	0.008**	0.005	0.007	0	0.003	0.004*	0.003	0.037***	0.032***	0.006**
	-0.002	-0.003	-0.002	-0.004	-0.004	-0.004	-0.004	-0.004	-0.005	-0.003	-0.003	-0.002	-0.003	-0.003	-0.004	(0.003)
post1*urban	-0.163***	-0.197***	-0.260***	-0.073	-0.094**	-0.039	-0.064*	-0.004	-0.026	-0.188***	-0.127***	-0.003	-0.105***	0.044	0.016	-0.116***
	-0.028	-0.042	-0.04	-0.045	-0.047	-0.038	-0.038	-0.04	-0.041	-0.045	-0.047	-0.058	-0.04	-0.053	-0.048	(0.028)
post2*urban	0.002	-0.081	-0.105	0.041	0.018	-0.033	-0.053	-0.016	-0.055*	0.014	-0.097***	-0.180***	-0.083***	0.01	0.078	0.159***
	-0.038	-0.067	-0.073	-0.033	-0.037	-0.034	-0.035	-0.028	-0.029	-0.056	-0.03	-0.068	-0.027	-0.046	-0.061	(0.020)
post3*urban	-0.058**	-0.190***	-0.161***	0.084**	0.072	-0.003	-0.006	-0.033	-0.047	-0.002	-0.115**	-0.141	-0.090*	-0.086*	-0.117**	0.155***
	-0.029	-0.062	-0.053	-0.042	-0.055	-0.045	-0.052	-0.036	-0.048	-0.072	-0.049	-0.091	-0.051	-0.045	-0.048	(0.019)
post1*coverage	-0.150***	-0.046	-0.055*	-0.249***	-0.284***	-0.339***	-0.373***	-0.446***	-0.449***	-0.432***	-0.261**	-0.335***	-0.335***	-0.210***	-0.207***	-0.108***
	-0.055	-0.046	-0.033	-0.089	-0.087	-0.075	-0.068	-0.044	-0.041	-0.045	-0.108	-0.117	-0.112	-0.05	-0.044	(0.041)
post2*coverage	0.107***	-0.092	-0.05	0.112***	0.085*	0.204***	0.164***	0.119*	0.143**	-0.173***	0.276***	0.317***	0.245***	0.089***	0.207***	-0.008
	-0.035	-0.06	-0.05	-0.043	-0.044	-0.05	-0.046	-0.071	-0.061	-0.044	-0.055	-0.073	-0.051	-0.031	-0.028	(0.042)
post3*coverage	-0.059*	-0.190***	-0.188***	-0.175***	-0.212***	-0.151**	-0.195***	-0.120*	-0.120*	-0.385***	-0.171***	-0.172**	-0.178***	-0.047*	-0.025	-0.270***
	-0.035	-0.044	-0.038	-0.053	-0.055	-0.066	-0.058	-0.07	-0.066	-0.054	-0.065	-0.08	-0.061	-0.026	-0.04	(0.051)
Observations	6,300	6,300	6,300	6,300	6,300	6,300	6,300	6,300	6,300	6,300	6,300	6,300	6,300	6,300	6,300	6,299

Table S9: Results from Model 5 (controlled interrupted time-series using a matched set of control districts; matching on region, coverage, and MI participation)

	BCG	HepB0	OPV0	DTP1	OPV1	DTP2	OPV2	DTP3	OPV3	M1	DTPb	M2	OPVb	TT1	TT2	Sessions
date_inyears	-0.025***	0.064***	0.009	-0.006	0.005	-0.011	0.001	-0.006	0.004	0.031**	-0.024	0.050**	-0.004	0.009	0.043***	0.012*
	-0.009	-0.018	-0.007	-0.009	-0.01	-0.01	-0.011	-0.01	-0.011	-0.014	-0.033	-0.022	-0.026	-0.01	-0.012	(0.007)
post1	0.011	0.018	-0.018	0.031	0.028	0.044**	0.029	0.021	0.008	0.022	0.123**	0.031	0.086*	-0.032	-0.028	0.008
	-0.017	-0.029	-0.015	-0.02	-0.025	-0.021	-0.022	-0.02	-0.023	-0.027	-0.061	-0.043	-0.05	-0.023	-0.024	(0.018)
post2	-0.016	-0.034	-0.048**	0.014	0.01	0.036**	0.029*	0.040**	0.038**	0.037	0.065	-0.170***	0.028	-0.040*	-0.042**	0.025*
	-0.022	-0.03	-0.019	-0.016	-0.018	-0.016	-0.017	-0.016	-0.018	-0.027	-0.055	-0.034	-0.044	-0.022	-0.02	(0.013)
post3	0	-0.051	-0.052***	0.009	-0.024	0.019	-0.014	0.007	-0.021	-0.034	0.072	-0.209***	0.003	0.007	-0.015	0.013
	-0.017	-0.036	-0.016	-0.015	-0.023	-0.017	-0.024	-0.017	-0.023	-0.038	-0.078	-0.068	-0.065	-0.017	-0.021	(0.015)
IMI*post1	0.052**	-0.002	0.028**	0.041	0.038	0.036	0.037	0.058***	0.056**	0.070*	0.081	0.073	0.085	-0.004	0.013	0.118***
	-0.026	-0.022	-0.014	-0.033	-0.035	-0.026	-0.024	-0.021	-0.023	-0.041	-0.062	-0.057	-0.059	-0.029	-0.022	(0.026)
IMI*post2	-0.001	0.057**	0.049***	-0.02	-0.026	-0.041**	-0.038**	-0.047**	-0.056***	-0.063**	-0.045	0.075**	-0.043	-0.037**	-0.049***	-0.044
	-0.024	-0.024	-0.016	-0.02	-0.022	-0.02	-0.019	-0.02	-0.02	-0.031	-0.033	-0.035	-0.028	-0.017	-0.012	(0.029)
IMI*post3	0.013	0.082***	0.061***	0.006	-0.001	0.003	0.001	0.014	0.001	-0.031	0.011	0.127**	0.016	0.012	-0.018	-0.010
	-0.025	-0.026	-0.018	-0.021	-0.023	-0.023	-0.022	-0.023	-0.022	-0.041	-0.042	-0.053	-0.039	-0.024	-0.016	(0.033)
date_inyears*IMI	-0.003	-0.01	0.004	-0.005	-0.001	-0.001	0.002	-0.001	0.007	0.039*	0.002	-0.016	0.003	-0.020**	0.003	0.005
	-0.01	-0.011	-0.007	-0.009	-0.01	-0.011	-0.009	-0.011	-0.009	-0.021	-0.016	-0.02	-0.014	-0.009	-0.007	(0.015)
IMI*post1*urban	-0.149***	-0.204***	-0.244***	-0.057	-0.078	-0.011	-0.033	0.047	0.024	-0.133***	-0.08	0.078	-0.048	0.042	0.038	-0.021
	-0.039	-0.047	-0.044	-0.051	-0.053	-0.042	-0.042	-0.042	-0.043	-0.043	-0.052	-0.069	-0.044	-0.053	-0.048	(0.039)
IMI*post2*urban	0.031	-0.046	-0.057	0.042	0.017	-0.049	-0.065*	-0.023	-0.060**	0.044	-0.092***	-0.141**	-0.077***	0.007	0.082*	0.136***
	-0.051	-0.078	-0.076	-0.036	-0.041	-0.035	-0.036	-0.026	-0.026	-0.045	-0.03	-0.063	-0.022	-0.035	-0.048	(0.030)
IMI*post3*urban	-0.038	-0.179**	-0.137*	0.108**	0.111*	0.025	0.036	-0.007	-0.008	0.006	-0.09	-0.11	-0.058	-0.071**	-0.092***	0.135***
	-0.056	-0.088	-0.074	-0.045	-0.06	-0.049	-0.062	-0.042	-0.063	-0.067	-0.056	-0.096	-0.059	-0.036	-0.036	(0.029)
IMI*post1*coverage	0.032	-0.035	0.115	0.093*	0.017	-0.039	-0.079	-0.212***	-0.187**	0.061	-0.145*	-0.039	-0.192**	-0.196***	0.021	-0.225***
	-0.047	-0.126	-0.074	-0.055	-0.058	-0.094	-0.092	-0.07	-0.08	-0.07	-0.086	-0.128	-0.085	-0.039	-0.062	(0.032)
IMI*post2*coverage	0.069	-0.272***	-0.099*	0.081	0.019	0.136**	0.098*	0.048	0.098	-0.211***	0.166**	0.412***	0.126*	0.02	0.247***	-0.203***
	-0.047	-0.075	-0.055	-0.05	-0.052	-0.059	-0.059	-0.074	-0.071	-0.08	-0.072	-0.074	-0.07	-0.048	-0.05	(0.032)
IMI*post3*coverage	0.249	-0.384***	-0.159	0.086	0.055	0.140**	0.129**	0.109*	0.182***	-0.098	0.103	0.267***	0.097	0.005	0.300***	-0.185
	-0.081	-0.138	-0.123	-0.057	-0.057	-0.069	-0.052	-0.06	-0.056	-0.096	-0.082	-0.092	-0.077	-0.038	-0.055	(0.033)
Observations	12,924	12,924	12,924	12,924	12,924	12,924	12,924	12,924	12,924	12,924	12,924	12,924	12,924	12,924	12,912	12,908

Table S10: Results from Model 6 (controlled interrupted time-series with matching by state, coverage, and MI participation)

	BCG	HepB0	OPV0	DTP1	OPV1	DTP2	OPV2	DTP3	OPV3	M1	DTPb	M2	OPVb	TT1	TT2	Sessions
date_inyears	-0.004	0.079***	0.031***	0.013	0.024**	0.012	0.024*	0.016	0.027**	0.086***	-0.015	0.039*	0.019	0.011	0.055***	-0.004
	-0.009	-0.019	-0.008	-0.009	-0.011	-0.011	-0.012	-0.013	-0.013	-0.011	-0.037	-0.022	-0.029	-0.008	-0.008	-0.009
post1	0.004	-0.018	-0.021	0.014	0.007	0.009	-0.006	-0.008	-0.022	-0.027	0.114*	0.028	0.056	-0.024	-0.036**	0.004
	-0.015	-0.033	-0.016	-0.019	-0.022	-0.024	-0.024	-0.023	-0.023	-0.026	-0.065	-0.043	-0.052	-0.019	-0.018	-0.015
post2	-0.016	-0.062**	-0.053***	0.007	0.002	0.023	0.015	0.02	0.014	-0.032	0.082	-0.041	0.015	-0.035**	-0.041***	-0.016
	-0.019	-0.03	-0.016	-0.016	-0.019	-0.019	-0.021	-0.022	-0.023	-0.02	-0.062	-0.038	-0.053	-0.018	-0.014	-0.019
post3	-0.006	-0.057	-0.055***	0.013	-0.012	0.018	-0.009	0.013	-0.008	-0.098***	0.115	-0.058	0.018	0.014	-0.023	-0.006
	-0.019	-0.039	-0.017	-0.015	-0.02	-0.018	-0.023	-0.021	-0.023	-0.029	-0.081	-0.049	-0.064	-0.013	-0.015	-0.019
IMI*post1	0.041**	0.03	0.025*	0.040*	0.043*	0.046*	0.053**	0.072***	0.066***	0.078***	0.079*	0.090*	0.097**	-0.021	0.018	0.041**
	-0.018	-0.023	-0.013	-0.023	-0.023	-0.024	-0.023	-0.017	-0.017	-0.026	-0.045	-0.052	-0.043	-0.023	-0.017	-0.018
IMI*post2	0.01	0.092***	0.049***	0.009	0.008	-0.009	-0.002	-0.01	-0.02	0.037**	-0.043***	-0.03	-0.022	-0.034***	-0.042***	0.01
	-0.019	-0.014	-0.012	-0.013	-0.012	-0.015	-0.013	-0.016	-0.013	-0.016	-0.015	-0.019	-0.016	-0.011	-0.01	-0.019
IMI*post3	0.006	0.079***	0.038**	0.009	0.001	0.005	0.002	0.007	-0.016	0.025*	-0.022	-0.032	0.001	-0.019	-0.029*	0.006
	-0.022	-0.02	-0.02	-0.016	-0.013	-0.019	-0.016	-0.019	-0.014	-0.015	-0.019	-0.02	-0.02	-0.014	-0.015	-0.022
date_inyears*IMI	-0.019	-0.018**	-0.012*	-0.018**	-0.019***	-0.015	-0.015*	-0.015*	-0.005	-0.01	-0.001	-0.004	-0.008	-0.011**	0.001	-0.019
	-0.009	-0.007	-0.006	-0.008	-0.007	-0.01	-0.009	-0.008	-0.006	-0.008	-0.008	-0.009	-0.008	-0.005	-0.005	-0.009
IMI*post1*urban	-0.051	-0.193***	-0.153***	0.061	0.07	0.154***	0.156***	0.241***	0.226***	0.016	0.05	0.248***	0.093	0.163***	0.186***	-0.051
	-0.043	-0.048	-0.043	-0.061	-0.063	-0.057	-0.057	-0.053	-0.051	-0.05	-0.072	-0.094	-0.062	-0.049	-0.046	-0.043
IMI*post2*urban	0.117*	-0.018	0.019	0.124**	0.126**	0.02	0.02	0.056*	0.033	0.195***	-0.04	-0.151**	-0.014	0.052	0.117***	0.117*
	-0.064	-0.084	-0.079	-0.053	-0.056	-0.048	-0.049	-0.034	-0.03	-0.037	-0.045	-0.071	-0.032	-0.033	-0.033	-0.064
IMI*post3*urban	0.098	-0.145	-0.047	0.228***	0.256***	0.160***	0.179***	0.121**	0.123**	0.129**	0.03	0.003	0.077	-0.016	0.016	0.098
	-0.069	-0.109	-0.098	-0.05	-0.062	-0.053	-0.065	-0.049	-0.062	-0.052	-0.072	-0.104	-0.07	-0.042	-0.032	-0.069
IMI*post1*coverage	0.07	-0.115	0.039	0.09	0.04	-0.062	-0.096	-0.341***	-0.280**	0	-0.162	-0.327**	-0.246**	-0.187***	0.037	0.07
	-0.082	-0.175	-0.099	-0.089	-0.083	-0.123	-0.126	-0.11	-0.109	-0.059	-0.111	-0.149	-0.1	-0.035	-0.081	-0.082
IMI*post2*coverage	0.165	-0.453***	-0.102	0.160**	0.125**	0.256***	0.228***	0.122*	0.198***	-0.324***	0.270***	0.308***	0.209**	0.131***	0.368***	0.165
	-0.08	-0.122	-0.08	-0.068	-0.063	-0.064	-0.064	-0.07	-0.069	-0.091	-0.101	-0.093	-0.089	-0.032	-0.076	-0.08
IMI*post3*coverage	0.339	-0.488**	-0.211	0.143*	0.147**	0.218***	0.259***	0.212***	0.331***	0.088	0.331***	0.494***	0.309***	0.230***	0.559***	0.339
	-0.135	-0.214	-0.177	-0.078	-0.065	-0.075	-0.06	-0.072	-0.06	-0.066	-0.1	-0.132	-0.088	-0.058	-0.072	-0.135
Observations	7,128	7,128	7,128	7,128	7,128	7,128	7,128	7,128	7,128	7,128	7,128	7,128	7,128	7,128	7,128	7,128

Table S11: Results from Model 7 (controlled interrupted time-series using non-contiguous control districts)

	BCG	HepB0	OPV0	DTP1	OPV1	DTP2	OPV2	DTP3	OPV3	M1	DTPb	M2	OPVb	TT1	TT2	Sessions
date_inyears	-0.001	0.040*	0.012	0.018	0.029*	0.001	0.011	-0.01	0	0.073***	0	-0.045	-0.034	0.017	0.015	-0.046***
	-0.015	-0.022	-0.018	-0.016	-0.015	-0.017	-0.016	-0.017	-0.016	-0.022	-0.029	-0.03	-0.027	-0.014	-0.013	(0.018)
post1	-0.034	-0.015	-0.039	-0.033	-0.039	0.015	0	0.031	0.018	0.019	0.039	0.062	0.045	-0.019	0.028	0.024
	-0.027	-0.041	-0.035	-0.027	-0.026	-0.025	-0.023	-0.023	-0.022	-0.063	-0.051	-0.051	-0.044	-0.021	-0.018	(0.019)
post2	-0.016	0	-0.014	-0.042	-0.047	-0.031	-0.032	-0.002	-0.004	-0.100**	-0.111**	0.021	0.007	-0.047**	0.005	0.045
	-0.04	-0.049	-0.043	-0.032	-0.03	-0.032	-0.028	-0.029	-0.028	-0.045	-0.044	-0.047	-0.042	-0.021	-0.019	(0.028)
post3	-0.01	-0.053	-0.056	-0.005	-0.036	0.009	-0.019	0.012	-0.014	-0.086**	-0.091	0.081	0.037	-0.023	-0.011	0.082**
	-0.037	-0.049	-0.044	-0.038	-0.037	-0.037	-0.035	-0.036	-0.033	-0.036	-0.062	-0.067	-0.06	-0.022	-0.019	(0.035)
IMI*post1	0.132***	0.054	0.079	0.176***	0.168***	0.130***	0.125***	0.095**	0.090**	0.109**	0.124**	0.166**	0.150**	-0.031	-0.035	0.099***
	-0.04	-0.064	-0.051	-0.046	-0.049	-0.043	-0.044	-0.043	-0.043	-0.048	-0.06	-0.072	-0.068	-0.038	-0.027	(0.037)
IMI*post2	-0.017	-0.014	-0.016	0.049	0.043	0.035	0.032	0	-0.007	0.02	0.052	-0.007	-0.03	-0.027	-0.102***	-0.093*
	-0.062	-0.093	-0.075	-0.053	-0.057	-0.055	-0.056	-0.053	-0.051	-0.038	-0.069	-0.072	-0.067	-0.037	-0.028	(0.052)
IMI*post3	0.063	0.093	0.075	0.055	0.04	0.04	0.031	0.028	0.019	0.03	0.045	0.024	0.002	0.063	0.017	-0.091
	-0.061	-0.1	-0.079	-0.059	-0.064	-0.062	-0.063	-0.06	-0.058	-0.042	-0.079	-0.083	-0.077	-0.038	-0.029	(0.059)
date_inyears*IMI	-0.033	-0.002	-0.002	-0.047*	-0.039	-0.027	-0.02	-0.005	0.005	0.016	0.025	0.021	0.033	-0.034*	0.031**	0.068**
	-0.024	-0.043	-0.033	-0.024	-0.026	-0.028	-0.027	-0.029	-0.025	-0.021	-0.034	-0.038	-0.035	-0.02	-0.012	(0.030)
IMI*post1*urban	-0.156***	-0.174***	-0.245***	-0.075	-0.097**	-0.041	-0.067*	-0.003	-0.026	-0.188***	-0.005	-0.129***	-0.106***	0.048	0.017	-0.116***
	-0.036	-0.047	-0.044	-0.048	-0.049	-0.038	-0.038	-0.041	-0.042	-0.045	-0.057	-0.047	-0.04	-0.056	-0.05	(0.029)
IMI*post2*urban	0.024	-0.03	-0.069	0.044	0.019	-0.032	-0.052	-0.015	-0.054*	0.013	-0.180***	-0.097***	-0.082***	0.031	0.092*	0.160***
	-0.049	-0.082	-0.086	-0.034	-0.039	-0.035	-0.035	-0.028	-0.029	-0.056	-0.069	-0.031	-0.027	-0.039	-0.055	(0.020)
IMI*post3*urban	-0.04	-0.156	-0.139*	0.094**	0.081	0.006	0.003	-0.029	-0.041	-0.002	-0.139	-0.114**	-0.088*	-0.061**	-0.102***	0.157***
	-0.052	-0.095	-0.08	-0.041	-0.054	-0.045	-0.053	-0.037	-0.05	-0.072	-0.093	-0.051	-0.052	-0.03	-0.031	(0.019)
IMI*post1*coverage	-0.137***	-0.014	-0.036	-0.246***	-0.280***	-0.336***	-0.371***	-0.445***	-0.448***	-0.432***	-0.335***	-0.261**	-0.335***	-0.201***	-0.196***	-0.105**
	-0.056	-0.063	-0.044	-0.089	-0.087	-0.075	-0.069	-0.045	-0.043	-0.045	-0.118	-0.109	-0.113	-0.047	-0.042	(0.041)
IMI*post2*coverage	0.093**	-0.125	-0.072	0.109**	0.082*	0.201***	0.161***	0.118*	0.142**	-0.173***	0.323***	0.279***	0.248***	0.079***	0.199***	-0.008
	-0.045	-0.078	-0.065	-0.045	-0.045	-0.051	-0.046	-0.071	-0.06	-0.044	-0.073	-0.055	-0.052	-0.028	-0.027	(0.043)
IMI*post3*coverage	-0.075	-0.214***	-0.214***	-0.181***	-0.218***	-0.154**	-0.197***	-0.122*	-0.123*	-0.385***	-0.173**	-0.171***	-0.178***	-0.068***	-0.037	-0.272
	-0.042	-0.065	-0.049	-0.055	-0.057	-0.069	-0.06	-0.072	-0.067	-0.054	-0.08	-0.065	-0.061	-0.024	-0.033	(0.051)
Observations	14,220	14,220	14,220	14,220	14,220	14,220	14,220	14,220	14,220	14,220	14,220	14,220	14,220	14,220	14,189	14,202

Table S12: Results from Model 8 (controlled interrupted time-series using non-contiguous control districts matched on coverage and MI participation)

	BCG	HepB0	OPV0	DTP1	OPV1	DTP2	OPV2	DTP3	OPV3	M1	DTPb	M2	OPVb	TT1	TT2	Sessions
date_inyears	-0.01	0.047**	0.007	0.009	0.016	-0.009	-0.001	-0.018	-0.011	0.060*	-0.044	0.033	-0.033	0.014	0.029**	-0.042**
	-0.015	-0.021	-0.017	-0.01	-0.011	-0.012	-0.011	-0.013	-0.013	-0.031	-0.027	-0.026	-0.024	-0.012	-0.013	(0.017)
post1	-0.024	0.001	-0.035	-0.032**	-0.028	0.014	0.005	0.036**	0.030*	0.038	0.055	0.035	0.04	-0.006	0.01	0.019
	-0.022	-0.034	-0.03	-0.016	-0.018	-0.019	-0.017	-0.018	-0.017	-0.052	-0.048	-0.043	-0.041	-0.02	-0.015	(0.018)
post2	-0.02	-0.007	-0.033	-0.048**	-0.046**	-0.026	-0.029	-0.012	-0.008	0.014	0.025	-0.064	0.011	-0.037**	-0.016	0.057**
	-0.033	-0.043	-0.04	-0.021	-0.021	-0.024	-0.021	-0.025	-0.023	-0.065	-0.042	-0.054	-0.037	-0.018	-0.018	(0.027)
post3	-0.029	-0.056	-0.067	-0.027	-0.042*	0.001	-0.019	0.007	-0.009	-0.137**	0.078	-0.190***	0.034	-0.022	-0.047**	0.091***
	-0.035	-0.05	-0.043	-0.022	-0.024	-0.027	-0.025	-0.027	-0.026	-0.059	-0.062	-0.061	-0.055	-0.019	-0.019	(0.030)
IMI*post1	0.110**	0.036	0.069	0.178***	0.165***	0.134***	0.129***	0.089**	0.078*	0.084**	0.185**	0.150*	0.168**	-0.066	-0.047*	0.107***
	-0.045	-0.075	-0.062	-0.04	-0.047	-0.042	-0.043	-0.044	-0.044	-0.039	-0.077	-0.081	-0.072	-0.045	-0.028	(0.030)
IMI*post2	-0.05	-0.05	-0.04	0.045	0.034	0.013	0.015	-0.011	-0.028	-0.123***	-0.015	0.02	-0.043	-0.045	-0.097***	-0.101**
	-0.064	-0.104	-0.087	-0.046	-0.054	-0.054	-0.055	-0.055	-0.055	-0.039	-0.074	-0.092	-0.067	-0.043	-0.028	(0.048)
IMI*post3	0.059	0.083	0.063	0.061	0.032	0.027	0.01	0.009	-0.017	0.04	0.024	0.119	-0.007	0.047	0.028	-0.102**
	-0.073	-0.124	-0.097	-0.051	-0.058	-0.061	-0.062	-0.061	-0.062	-0.046	-0.085	-0.105	-0.076	-0.045	-0.031	(0.051)
date_inyears*IMI	-0.022	0.006	0.016	-0.038*	-0.03	-0.016	-0.009	0.009	0.022	0.035	0.013	-0.02	0.027	-0.029	0.024	0.054**
	-0.029	-0.05	-0.04	-0.021	-0.026	-0.027	-0.026	-0.029	-0.026	-0.022	-0.039	-0.044	-0.035	-0.022	-0.015	(0.028)
IMI*post1*urban	-0.282***	-0.315***	-0.319***	-0.176***	-0.217***	-0.154***	-0.192***	-0.104**	-0.152***	-0.249***	-0.289***	-0.151***	-0.265***	-0.123*	-0.142**	-0.302***
	-0.042	-0.063	-0.05	-0.047	-0.046	-0.036	-0.039	-0.044	-0.041	-0.039	-0.038	-0.04	-0.031	-0.068	-0.064	(0.028)
IMI*post2*urban	-0.098**	-0.191**	-0.186***	-0.03	-0.088**	-0.117***	-0.163***	-0.111***	-0.174***	-0.158**	-0.257***	-0.243***	-0.248***	-0.110**	0.003	0.004
	-0.042	-0.075	-0.068	-0.036	-0.041	-0.038	-0.037	-0.035	-0.033	-0.062	-0.038	-0.09	-0.038	-0.048	-0.083	(0.024)
IMI*post3*urban	-0.157***	-0.330***	-0.274***	0.026	-0.042	-0.085	-0.131***	-0.128***	-0.194***	-0.071	-0.232***	-0.133	-0.218***	-0.215***	-0.282***	-0.005
	-0.049	-0.1	-0.09	-0.043	-0.048	-0.052	-0.048	-0.043	-0.043	-0.095	-0.073	-0.113	-0.065	-0.038	-0.036	(0.024)
IMI*post1*coverage	0.099*	0.042	0.049	-0.027	-0.053	-0.14	-0.166	-0.318***	-0.286***	-0.259***	-0.157	-0.258	-0.268*	0.017	-0.005	-0.080
	-0.058	-0.078	-0.056	-0.103	-0.101	-0.107	-0.106	-0.084	-0.079	-0.053	-0.141	-0.166	-0.148	-0.059	-0.061	(0.048)
IMI*post2*coverage	0.278***	-0.085	-0.011	0.218***	0.207***	0.370***	0.334***	0.287***	0.333***	0.102*	0.431***	0.504***	0.404***	0.285***	0.346***	0.113**
	-0.058	-0.103	-0.085	-0.06	-0.066	-0.055	-0.057	-0.079	-0.072	-0.061	-0.074	-0.105	-0.076	-0.042	-0.058	(0.054)
IMI*post3*coverage	0.241	-0.206**	-0.049	0.013	0.04	0.087	0.084*	0.077	0.143***	-0.046	0.038	0.132**	0.063	0.095**	0.238***	0.037
	-0.062	-0.101	-0.088	-0.053	-0.063	-0.064	-0.051	-0.059	-0.049	-0.048	-0.076	-0.064	-0.067	-0.04	-0.051	(0.045)
Observations	12,564	12,564	12,564	12,564	12,564	12,564	12,564	12,564	12,564	12,564	12,564	12,564	12,564	12,564	12,533	12,547

References

[1] J. L. Bernal, S. Cummins, and A. Gasparrini, “Interrupted time series regression for the evaluation of public health interventions: a tutorial,” *Int. J. Epidemiol.*, p. dyw098, Jun. 2016, doi: 10.1093/ije/dyw098.

[2] J. L. Bernal, S. Cummins, and A. Gasparrini, “The use of controls in interrupted time series studies of public health interventions,” *International Journal of Epidemiology*, vol. 47, no. 6, pp. 2082–2093, Dec. 2018, doi: 10.1093/ije/dyy135.

[3] S. Iacus, G. King, and G. Porro, *CEM: Coarsened Exact Matching Software*. .
